# Selective C_70_ encapsulation by a robust octameric nanospheroid held together by 48 cooperative hydrogen bonds

**DOI:** 10.1038/ncomms15109

**Published:** 2017-05-10

**Authors:** Grzegorz Markiewicz, Anna Jenczak, Michał Kołodziejski, Julian J. Holstein, Jeremy K. M. Sanders, Artur R Stefankiewicz

**Affiliations:** 1 Laboratory of Functional Nanostructures, Faculty of Chemistry, Adam Mickiewicz University in Poznań, Umultowska 89b, 61-614 Poznań, Poland; 2 Laboratory of Functional Nanostructures, Centre for Advanced Technologies, Adam Mickiewicz University, Umultowska 89c, 61-614 Poznań, Poland; 3 Faculty of Chemistry and Chemical Biology TU Dortmund, Otto-Hahn-Strasse 6, D-44227 Dortmund, Germany; 4 Department of Chemistry, University Chemical Laboratory, Lensfield Road, Cambridge CB2 1EW, UK

## Abstract

Self-assembly of multiple building blocks via hydrogen bonds into well-defined nanoconstructs with selective binding function remains one of the foremost challenges in supramolecular chemistry. Here, we report the discovery of a enantiopure nanocapsule that is formed through the self-assembly of eight amino acid functionalised molecules in nonpolar solvents through 48 hydrogen bonds. The nanocapsule is remarkably robust, being stable at low and high temperatures, and in the presence of base, presumably due to the co-operative geometry of the hydrogen bonding motif. Thanks to small pore sizes, large internal cavity and sufficient dynamicity, the nanocapsule is able to recognize and encapsulate large aromatic guests such as fullerenes C_60_ and C_70_. The structural and electronic complementary between the host and C_70_ leads to its preferential and selective binding from a mixture of C_60_ and C_70_.

## Abstract (web summary)

Individual hydrogen bonds are weak, so self-assembling multiple components via hydrogen bonding is a significant challenge. Here the authors report a robust, enantiopure nanocapsule held together by 48 cooperative hydrogen bonds, and use it for the selective binding of C_70_.

Multimembered, three-dimensional chemical entities such as cages or capsules are complex architectures that can be constructed using self-assembly processes where building blocks are held together by dynamic covalent or non-covalent bonds. The continuing development of supramolecular chemistry and the improved understanding of its underlying assembly processes have led to the synthesis of increasingly complex and sophisticated three-dimensional structures such as nanotubes^1^, cages^2^,^3^ and capsules^4^,^5^,^6^. There is a close relationship between the structure of a given architecture and its functionality, as in natural systems, and chemists have exploited this in the design of functional structures that possess desirable properties and that may be used inter alia to build molecular machines^7^.

Many viral genomes are hosted within hollow spherical protein capsules: self-assembly of these viral capsid is a complex process involving dynamic interactions between the participating protein subunits^8^. Despite the robust nature of the spherical capsid, achieved through number of conformational rearrangements, its dynamic nature, required to perform biological functions, is still preserved^9^.

The enclosed and protected spaces within capsules and cages are of particular interest because of their potential for selective encapsulation^2^,^10^ and catalysis by inclusion, examples being the ability to neutralize explosives^11^ or to separate enantiomers^12^. An established pathway to spherical structures involves the careful design and synthesis (often multi-stage) of an organic ligand with precisely spaced functional groups. Nanocapsules can be produced via reversible covalent bonds such as disulfide^13^,^14^, boronic esters^15^,^16^ and imines^17^,^18^,^19^,^20^,^21^,^22^ or supramolecular interactions such as coordinative bonds^23^,^24^,^25^,^26^ and hydrogen bonding^27^,^28^,^29^,^30^,^31^, the last being exemplified in the present work.

The vast majority of hydrogen-bonded capsules are dimeric species which are constructed and/or exist only in the presence of stabilising template molecules^10^,^32^. The formation of multimembered architectures via hydrogen bonds is much more challenging and rare, in part due to the potentially prohibitive entropic cost of creating large ordered objects^30^,^33^,^34^. Furthermore, hydrogen bonds are by nature dynamic linkages so self-assembled nanocapsules based on this interaction are generally fragile, particularly in solution at high temperatures or in the presence of a disruptive entity such as a base. Therefore, the use of chemical self-assembly process in the efficient generation of highly stable nanocapsular and multimembered objects with preserved function in selective guest binding has not been much explored.

Taking inspiration from natural systems we report here the discovery of a newly designed multimembered nanocapsule that is formed as a product of a self-assembly process in a nonpolar solvent. The capsule is based on the generation of 48 hydrogen bonds between 8 identical enantiomerically pure building blocks that consists of an aromatic ring with three attached amino acid residues acting as both H-bond acceptors and donors. This design of the molecular substrate has provided a supramolecular nanocapsule with a precisely defined size and shape as well as an outstanding thermal and chemical stability in solution. In contrast to the previously described system based on the same central aromatic platform^32^, the formation of the capsule is independent of the presence of stabilizing template molecules. Thanks to small pore sizes, large internal cavity and sufficient dynamicity, the presented nanocapsule has been found to selectively recognize and encapsulate large aromatic guests such as fullerenes C_60_ and C_70_. The selective encapsulation of the latter is particularly attractive, since their large scale separation is difficult^35^.

## Results

### Synthesis

**Figure 1:**
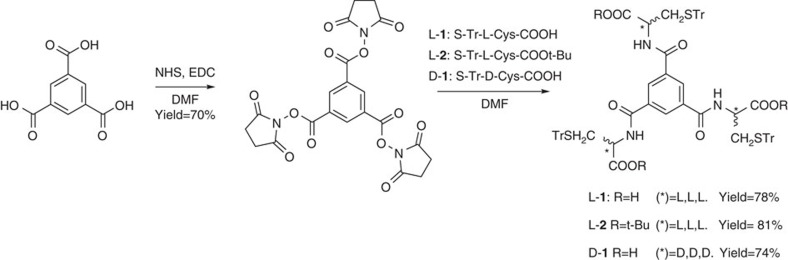
Building block synthesis. The synthesis of enantiomerically pure amino acid functionalised compounds employed in the nanocapsule formation, (Tr=trityl).

The synthesis of L-**1,** D-**1** and L-**2** followed a literature procedure^13^ involving reaction of an activated ester of benzene-1,3,5-tricarboxylic acid with the corresponding trityl-protected cysteine (Fig. 1, See Supplementary Methods section for details). In each case the two step procedure gave enantiomerically pure materials in high yields. It became obvious subsequently that the trityl group plays significant role in the generation of a three-dimensional architecture. The presence of this protecting group on the cysteine side chains was expected to enhance both the solubility and the stability of the hydrogen-bonded assembly, as a consequence of creation of a more nonpolar environment, which would raise the p*K*_a_ in a manner that is reminiscent of carboxylic groups in enzyme active sites^36^. Additionally, the robust crystallization properties of many compounds with trityl groups have led to the suggestion that their presence may boost the chances to obtain good quality crystals for X-ray analysis^37^.

### Solid state characterization of the nanocapsule

**Figure 2:**
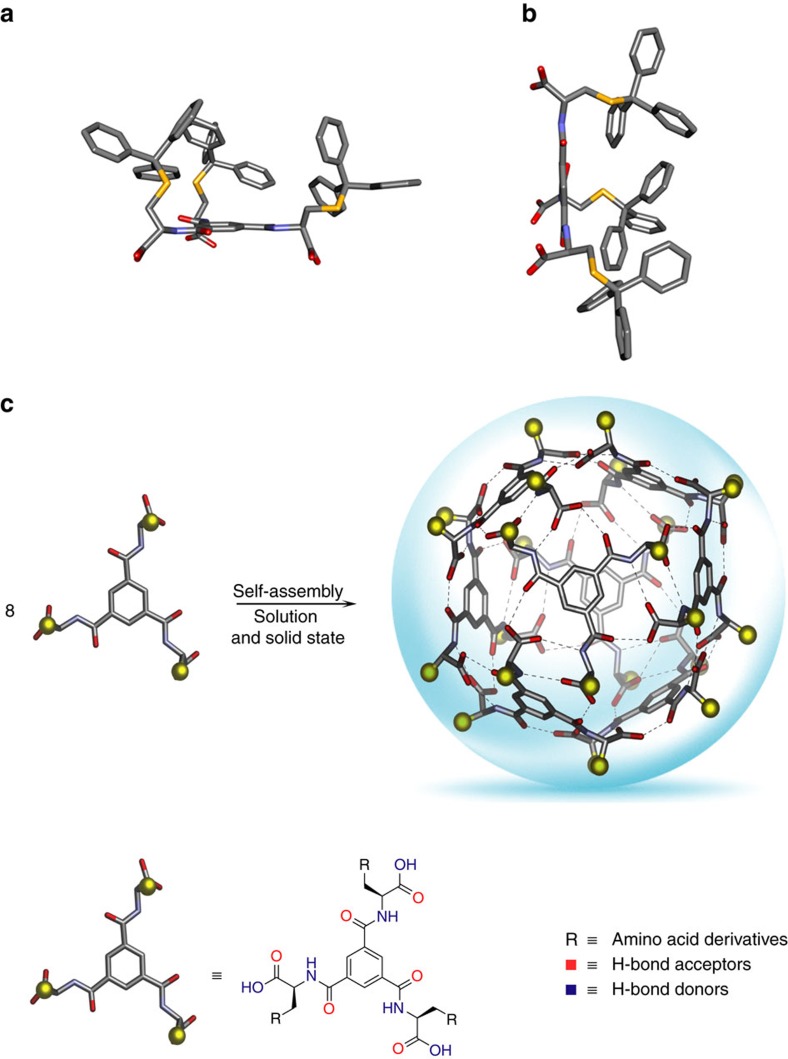
Solid state characterization of the nanocapsule. (**a**,**b**) The molecular species found in the lattice of L-**1**, as crystallized from chloroform; red=O, yellow=S and blue=N. Disorder and hydrogen atoms have been removed for clarity. (**c**) Self-assembly of a new supramolecular nanocapsule build up from 8 molecules of compound L-**1** via formation of 48 hydrogen bonding interactions. Yellow spheres represent trityl groups of the cysteine moieties.

Slow evaporation of chloroform solution of L-**1** resulted in the isolation of blocked-shaped light yellow crystals. Structural confirmation for L-**1** was provided by single-crystal X-ray crystallographic analysis. The compound L-**1** crystallizes in the tetragonal space group P4_**2**_2_**1**_2. The analysis was complicated by disorder of trityl groups near the crystallographic twofold axis (special positions). The key to a successful refinement was utilization of stereochemical restraints for the L-**1** structure, which were generated by the GRADE programme^38^. This macromolecular refinement technique has been adapted to be used in the programme SHELXL (refs 39, 40). We have already used this methodology for other supramolecular structures^41^, and it was found to drastically increase the robustness of the refinement, especially when combined with the new rigid bond restraint in SHELXL (RIGU)^42^. The programme ShelXle (ref. 43), which supports the macromolecular residue grouping, was used as a Graphical User Interface whereas the DSR programme was employed to model disordered moieties^44^. As shown in Fig. 2a,b, the crystal structure revealed that all the aromatic rings of the trityl groups are situated on the same side of the 1,3,5-benzene core, despite the steric hindrance, directing the three carboxylate groups in the opposite direction. Further analysis of the crystal structure reveals that L-**1** assembles through complementary and metrically matched hydrogen bonds to form an octameric supramolecular nanocapsule (Fig. 2c).

**Figure 3:**
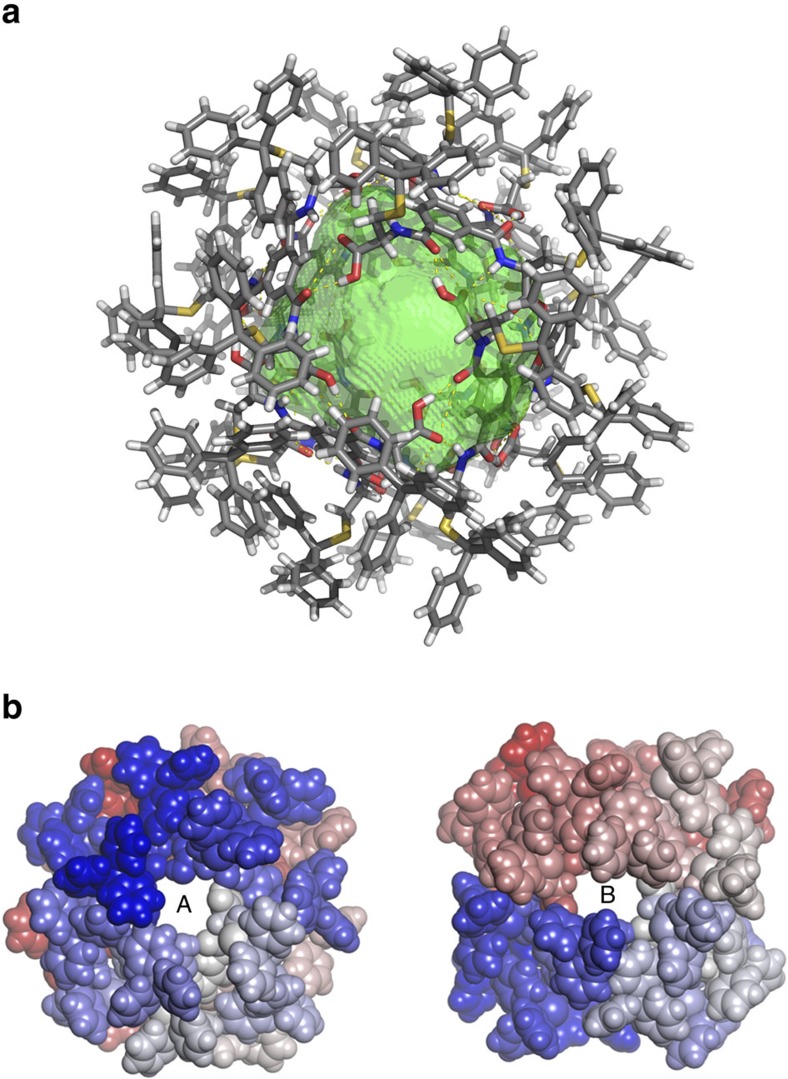
Internal cavity and the pores found in nanocapsule crystals. (**a**) The crystal structure of nanocapsule based on L-**1**; red=O, yellow=S and blue=N. Disorder has been removed for clarity. The encapsulated void volume of 1,719 Å^3^ is shown as 50% transparent green isosurface. (**b**) Space filling representations of the nanocapsule derived from L-**1** showing two types of pores A and B.

The data collection of the L-**1** nanocapsule structure posed typical difficulties observed for complex supramolecular structures with large percentage of void space containing unordered chloroform solvent molecules. Crystals were extremely volatile and decomposed or cracked within a couple of a few seconds after being taken out of the mother liquor. We attributed this phenomenon to the rapid loss of chloroform, which was used as crystallization solvent. The asymmetric unit contains two L-**1** molecules and the full nanocapsule is generated by crystallographic symmetry elements. The point group symmetry of the capsule in the crystal structure is D_2_ after Schoenflies (222 after Hermann-Mauguin). The cavity volume was calculated to be 1,719 Å^3^ (1.7 nm^3^) using a 3.0 Å probe in the void space calculation using VOIDOO (Fig. 3a)^45^. To prevent the probe from ‘escaping' the inner sphere through the large pores, the default probe radius of 1.4 Å (water) was increased to 3.0 Å. This results in smaller calculated volumes compared to using the default probe size, but provides the additional information that all molecules with diameter of 2.9 Å or smaller can penetrate the capsule. In the solid state no chloroform site could be identified inside the capsule. We assume that solvent molecules, are dynamically disordered inside the capsule and therefore do not produce a distinct signal in the X-ray measurement.

The external surface of the octameric nanocapsule based on L-**1** is not fully enclosed. As shown in Fig. 3b, the supramolecular assembly contains six pores of two types: A (four, each with diameter of 7.33 Å × 7.40 Å) and B (two, each with diameter 7.37 Å × 8.75 Å). The A-type pores are located along the equator of the nanocapsule, whereas B-type pores are placed at the ‘poles' of the assembly.

### Solution studies

**Figure 4:**
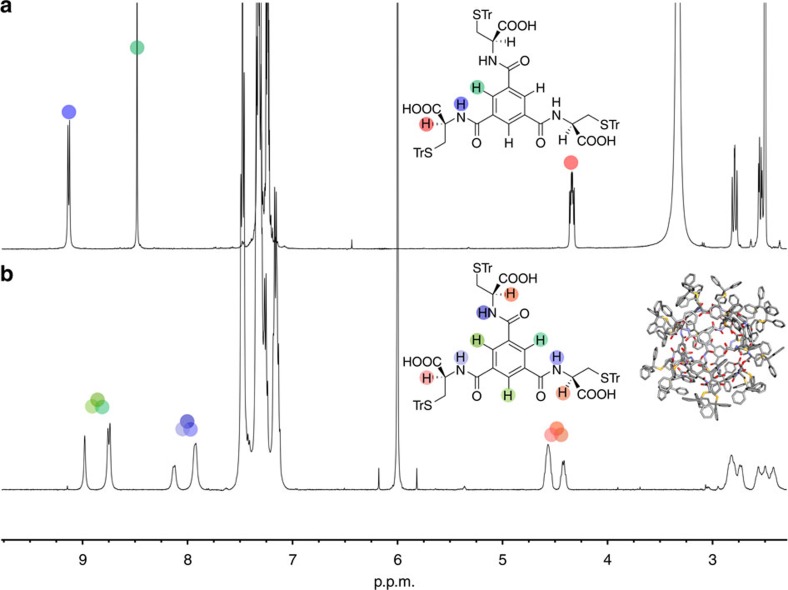
^1^H NMR (500 MHz) spectra at ambient temperature of L-1. Recorded in: (**a**): DMSO-*d*_6_, (**b**): TCE-*d*_2_.

Nuclear magnetic resonance (NMR) spectroscopic analyses in solution were in agreement with the solid-state data, confirming that the nanocapsule is retained in solution. Based on our earlier precedent of supramolecular nanotubes^1^, we investigated the NMR properties of L-**1,** D-**1** and L-**2** in different solvents. The ^1^H NMR spectra in DMSO-*d*_6_, an exceptionally strong H-bond acceptor, are unremarkable, displaying the expected *C*_3_ symmetry (L-**1** Fig. 4a, for D-**1** see Supplementary Fig. 2). This solvent completely disrupts any inter- (or intra-) molecular H-bonds, creating a solvated monomer in which any conformational interconversions are sufficiently rapid to lead to the expected *C*_3_ symmetry on the NMR chemical shift timescale.

The NMR spectrum of L-**1**, is significantly different in 1,1,2,2-tetrachloroethane (TCE-*d*_2_), a solvent that does not disrupt intermolecular H-bonding (Fig. 4b, for D-**1** see Supplementary Fig. 7). Now the spectrum is consistent with the effective *C*_*2*_ symmetry (ignoring disorder) of the octameric species seen in the lattice of the crystal obtained from chlorinated solvents, implying that the octamer is indeed preserved in TCE-*d*_2_ solution; the apparent 2:1 intensities ∼4.5 and 8.0 p.p.m. are due to accidental coincidences at ambient temperature which are removed on heating or cooling (see below). Analogous results were observed in the ^13^C NMR spectra of L-**1** in DMSO-*d*_*6*_ and TCE-*d*_*2*_, which reveal the same symmetry properties as those obtained by the ^1^H NMR analysis (see Supplementary Figs 9,10 and 12). The symmetry of the nanocapsules exhibited in these spectra corresponds to eight equivalent monomers, each having three non-equivalent functional arms.

The affinity for self-recognition (narcissistic self-sorting) between the nanocapsule subunits presenting opposite chirality at the α-carbon (L-**1** and D-**1**) has been investigated. The ^1^H NMR spectrum of an equimolar mixture of both molecules (L-**1** and D-**1**) in TCE-*d*_*2*_ was compared to that containing only L-**1**. Both spectra were found to be essentially identical without shifts and/or appearance of any new peaks (Supplementary Fig. 27a). This observation implies that the subunit, composed of either all-L- or all-D-amino acids is dictating the chirality of the assembly, leading in effect to the exclusive formation of the enantiopure nanocapsule (in contrast to the corresponding experiment in the Mastelerz *et al. *octameric capsule system, ref. 31). It also demonstrates that the homochiral nanocapsule is thermodynamically the most stable accessible structure. This result was further supported by the circular dichroism spectroscopy studies where equimolar mixture of all-L- and all-D subunits self-sort to give the nanocapsule in racemic form (Supplementary Fig. 27b). Since the enantiomerically pure capsules are formed from both L-**1** and D-**1**, we focused our further analysis on L-**1** which derive from natural cysteine isomer.

To explain the splitting effect observed, in both ^1^H and ^13^C NMR spectra, detailed analysis of the crystal structure was performed. Due to the structural features of the nanocapsule (four equatorial and two pairs of two polar building blocks), each arm of the monomer is located in a slightly different environment. This effect is most apparent when analysing the solid-state structure, where significant differences in the hydrogen bonding distances between functional arms in the monomer are observed (Supplementary Fig. 26). The differences in distances of hydrogen bonds directly affect their strength and will have an impact on the chemical shifts found in both ^1^H and ^13^C NMR spectra. This is visible when looking at the hydrogen bonding distances between aromatic central core protons and the carbonyl groups. In this case, two distances are found to be very similar (2.319 and 2.301 Å), while the third is significantly larger (2.423 Å). The proton in the latter case experiences a weaker shielding effect as observed in the NMR spectrum. The opposite H-bond distance desymmetrisation effect was observed for the amide N-H groups. Of the three amide protons, one is located substantially closer to the nearest H-bonding acceptor, leading to increased H-bond energy and deshielding of this proton signal. Both effects are fully consistent with the NMR spectra presented in Fig. 3b and S12 (see ESI). The higher order steric effects seen in the crystal structure (Supplementary Fig. 26) are unfortunately quenched in the solution studies by dynamic breathing motions of the capsule. The splitting of the different proton resonances of the monomer into three groups in 1:1:1 ratio, consistent with three-fold symmetry, is also clearly evident in the EASY-ROESY spectrum, where no exchange peaks are observed, suggesting that each of eight monomers of the nanocapsule is effectively equivalent (Supplementary Fig. 20). As a control, the NMR spectrum of L-**2** in TCE-*d*_*2*_ is simple and conventional as expected because the ester group is unable to generate the hydrogen bonds required for formation of the supramolecular nanocapsule (Supplementary Fig. 8).

**Figure 5:**
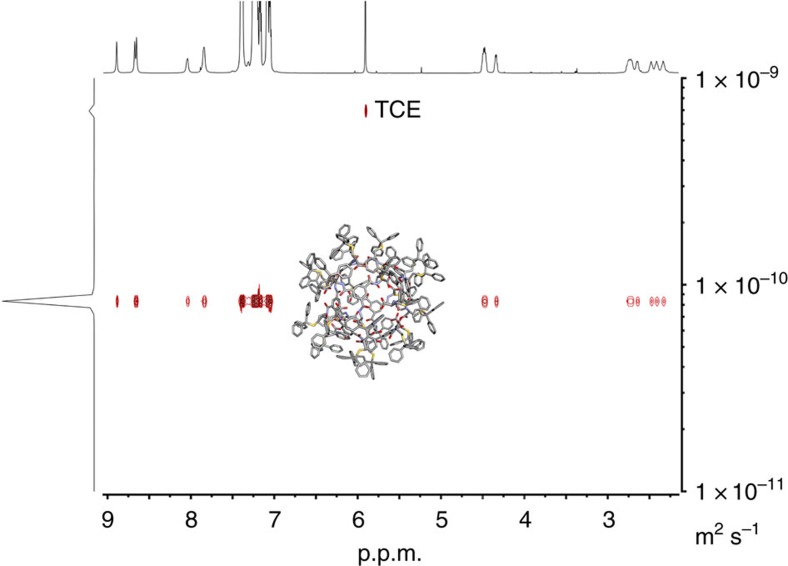
Diffusion ordered spectroscopy NMR spectrum (700 MHz TCE-*d*_2_). The diffusion coefficient of L-**1** is calculated to be 8.31 × 10^−11^ (m^2^ s^−1^).

The formation of unique and discrete supramolecular octameric nanocapsules from either L-**1** and D-**1** is further supported by diffusion ordered spectroscopy experiments (Fig. 5 for L-1, for D-1 see Supplementary Fig. 22). Using the Stokes-Einstein equation, the solvodynamic diameter of the nanocapsules has been found to be 18.2 Å (observed diffusion coefficients 8.31 and 8.20 × 10^−11^ (m^2^ s^−1^) for L-**1** and D-**1**, respectively), which is consistent with the diameters provided by single-crystal X-ray crystallographic analysis of L-**1** (19.4 Å along *X* axis and 16.2 Å along *Y* axis without trityl moieties, respectively and 29.4 and 25.5 Å with them). In contrast, the solvodynamic diameter for molecule L-**2**, which is unable to form capsular assembly has been found to be 10.59 Å (observed diffusion coefficient 1.42 × 10^−10^ (m^2^ s^−1^), see Supplementary Fig. 23).

### Thermodynamic stability of the nanocapsule

**Figure 6:**
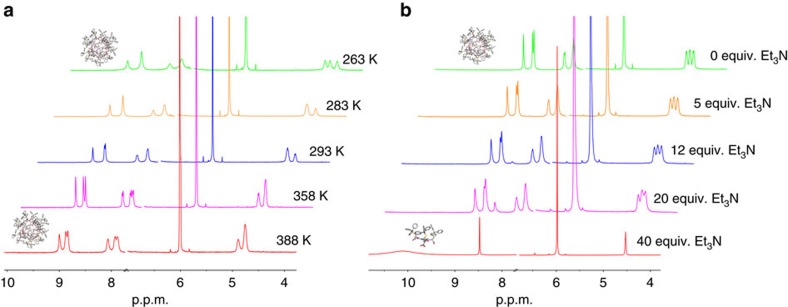
Thermodynamic stability of the nanocapsule. (**a**) VT NMR (500 MHz TCE-*d*_*2*_) spectra of compound L-**1**. (**b**) Base (Et_3_N) titration NMR spectra (500 MHz TCE-*d*_*2*_) of compound L-**1** recorded at 318 K.

We next tested the thermodynamic stability of the nanocapsule with VT NMR and base (Et_3_N) titration experiments. As shown in Fig. 6a the supramolecular structure is stable over a wide range of temperatures (from −10 °C up to 105 °C), exceptional behaviour for a purely H-bonded supramolecular oligomer. Furthermore, the supramolecular structure was also resistant to base (Fig. 6b). In principle, removal of the carboxylic acid protons should prevent H-bonding and thus lead to decomposition of the capsule, but more than 40 equiv. of triethylamine (1.66 equiv. per carboxyl group) at 318 K were required to produce a spectrum showing the *C*_3_ symmetry of the monomeric anion. The stability of this capsule clearly derives from highly co-operative hydrogen bonding interactions between donor and acceptor groups in neighbouring monomers. In principle, abolition of one hydrogen bond by deprotonation might be expected to lead to loss of that monomer unit from the capsule, but the enthalpic cost of rupturing additional H-bonds would be very large. This reinforcement of weak interactions by geometrical constraints is reminiscent of that observed in other systems such as the trefoil knot reported by one of us^46^ and Moore's recent kinetically-trapped tetrahedral cages^47^.

### Host–guest chemistry and selective binding of fullerenes

The nanocapsule of L-**1** presents an internal cavity suitable for encapsulating guest molecules through supramolecular interactions. We anticipated that the presence of large pores (the largest sphere that could freely pass through these pores would have a van der Waals radius of ∼4 Å) combined with a huge internal void cavity (1,719 Å^3^) might allow the free penetration of the capsule by relatively small molecules such as solvents, while guest molecules with diameter bigger than 8 Å may be considered for binding studies inside the capsule. We reasoned that the aromatic surfaces lining the cavity of the nanocapsule might lead to binding of large preferentially spherical aromatic guests. After topological, structural and electronic analysis of both the nanocapsule and potential guests, C_60_ and C_70_ fullerenes were selected for the binding studies. The use of supramolecular receptors for fullerene separation in solution has emerged as an attractive alternative to often tedious, energy- and time-consuming chromatographic techniques, since it allows potential selectivity, does not require specific equipment and ideal recyclable hosts can be designed. Since both fullerenes C_60_ and C_70_ are devoid of hydrogen atoms, the most convenient way to study their binding inside the nanocapsule is ^13^C NMR analysis. The ^13^C NMR resonance signal of free C_60_ in TCE-*d*_*2*_ was recorded at 142.85 p.p.m. (Supplementary Fig. 13). During the addition of the L-**1** nanocapsule to a saturated solution of C_60_ in TCE-*d*_*2*_, a new upfield signal appeared at 140.77 p.p.m., originating from the host–guest complex (|*δ*_fullerene_−*δ*_guest_|=2.08 p.p.m., Supplementary Fig. 13). The large upfield shift indicates that C_60_ resides within the shielded interior of the cavity composed of 8 molecules of L-**1** and it is in good agreement with values observed for the previously reported fullerene receptor based on double-calix[5] arene^48^ and our supramolecular nanotube^1^.

**Figure 7:**
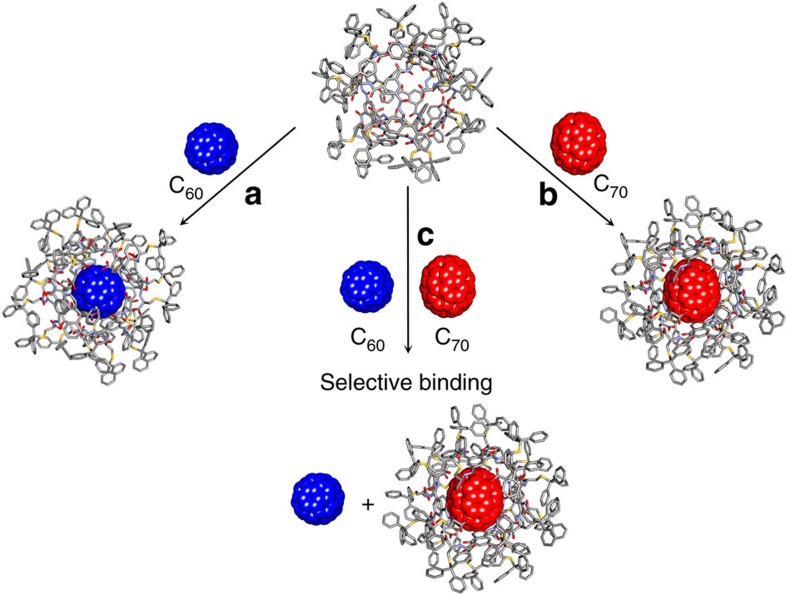
Fullerenes sequestration. Schematic representation of the binding studies between the multimembered nanocapsule based on L**-1** and (**a**) C_60_ fullerene. (**b**) C_70_ fullerene (**c**) selective encapsulation of C_70_ from the equimolar mixture of C_60_/C_70_ fullerenes and the nanocapsule.

A similar encapsulation result was obtained for C_70_ fullerene, whose five ^13^C NMR signals (150.32, 147.76, 146.50, 145.00 and 130.53 p.p.m.) were also found to be shifted towards higher magnetic field by about 2 p.p.m. when mixed with the nanocapsule (Supplementary Fig. 14); intriguingly more than five shifted signals are observed (8 in total) presumably due to the reduced effective symmetry experienced within the chiral cavity of the nanocapsule. From the diameters calculated on the basis of the X-ray structures of both the nanocapsule (15.9 × 16.9 Å) and fullerenes C_60_ (10.3 × 10.3 Å) and C_70_ (10 × 11.3 Å) we anticipated that in both cases 1:1 complex are formed (Fig. 7a,b). The binding process between fullerene guests (C_60_ and C_70_) and the L-**1** nanocapsule was followed by ultraviolet–visible titration in TCE at 26 °C (Supplementary Fig. 28, see Supplementary Methods for details); both C_60_ and C_70_ were observed to form 1:1 host–guest complexes with the L-**1** nanocapsule in TCE. The guest binding strength was found to be 7.6 × 10^1^ M^−1^ and 1.0 × 10^3^ M^−1^ for C_60_ and C_70_, respectively. The encapsulation of fullerene molecules must involve temporary opening of the L-**1** nanocapsule to allow guest entry followed by the receptor's reassembly. To further illustrate the potential of this system, we employed the two fullerene guests together in a competition experiment (Fig. 7c). Based on the binding studies with C_60_ and C_70_ inside nanocapsule derived from L-**1**, an experimental set-up was designed to use the latter in the selective binding and separation of C_60_ and C_70_. The binding study between equimolar amounts of the capsule with C_60_ and C_70_ fullerenes was followed by ^13^C NMR spectroscopy and showed preferential formation of the complex with C_70_ over that of the C_60_ (signals indicative of the encapsulated C_70_ between *δ*=148–142 p.p.m. and 128 p.p.m., see Supplementary Fig. 15). We infer that the difference in the shape of the guest molecules might be the origin of the observed selectivity, as the host–guest interactions are very similar in both cases. Both fullerenes are based on the extended *π*-system and contain carbon atoms with *sp*^2^ hybridization, however, unlike the ideally spherical C_60_, C_70_ has ellipsoidal shape. Since the supramolecular host is also slightly elongated along one axis, it seems that there is a perfect electronic and more importantly structural complementarity between the C_70_ and the nanocapsule leading to the guest selectivity through more effective and stronger interactions (*π*-stacking) between them.

## Discussion

In conclusion, we describe for the first time the generation of a new capsid-like purely organic spheroidal assembly consisting of eight monomers analogous to the proteins that form spherical virus capsids. The self-assembly process depends on the formation of 48 hydrogen bonds between carboxyl and amine moieties of adjacent molecules. This remarkably complex three-dimensional architecture discovered in the solid state persists also in chlorinated solvents. We believe this is the largest purely synthetic and enantiopure spheroid formed through co-operative non-covalent interactions. The cooperativity of hydrogen bonding is imposed by the geometry, conferring outstanding temperature stability and resistance to base. Utilization of the self-assembly process in the generation of efficient and specific receptors for selected guests is an important part of supramolecular chemistry with potential applications in nanotechnology and in material and separation sciences. We demonstrated that this new nanocapsule acts as selective receptor for fullerenes C_60_ and C_70_ and facilitates the selective binding and extraction of latter one from the mixture of two. Most of the alternative methods for fullerene separation and purification are only selective towards the major component C_60_^49^; examples in the literature in which C_70_ are preferred are scarce. Although a few examples of C_70_ and C_60_ fullerenes and receptors have been reported, they were mainly based on the dimeric structures^50^, or metal-ligand coordinative bonds^49^,^51^,^52^,^53^,^54^. The presented system consist on purely organic and multimembered spheroidal architecture and for reasons of synthetic economy it provides a major advantage since only one molecule is needed to generate selective receptor for fullerenes. This discovery represent a major advance in the field of hydrogen-bonded assemblies, not only through their synthesis, but also by being versatile in their room for structural variation and possible side chain decoration on the surface in a manner reminiscent of phage display. Despite the enormous progress in the research on hydrogen-bonded structures, our results present a new perspective on the generation of highly dynamic supramolecular system where the repetition of the same subunits provide access to topologically complex functional nanostructure. The large enclosed free volume offered by this supramolecular assembly has also potential utility for catalysis and transport that we intend to explore.

### Data availability

Crystallographic data including structure factors have been deposited with CCDC no. 1469874. Copies of the data can be obtained free of charge at the CCDC website http://www.ccdc.cam.ac.uk/structures. All other data is available from the authors upon reasonable request.

## Additional information

**How to cite this article:** Markiewicz, G. *et al*. Selective C_70_ encapsulation by a robust octameric nanospheroid held together by 48 cooperative hydrogen bonds. *Nat. Commun.* **8,** 15109 doi: 10.1038/ncomms15109 (2017).

**Publisher's note**: Springer Nature remains neutral with regard to jurisdictional claims in published maps and institutional affiliations.

## Supplementary Material

Supplementary Information Supplementary figures, supplementary tables, supplementary methods and supplementary references.
