# Supplementary material for: Selective C70 encapsulation by a robust octameric nanospheroid held together by 48 cooperative hydrogen bonds
Source: Nat Commun. 2017 May 10;8:15109. doi: 10.1038/ncomms15109 (PMC5436139; doi:10.1038/ncomms15109)
Supplement: Supplementary Information — Supplementary figures, supplementary tables, supplementary methods and supplementary references. [file ncomms15109-s1.pdf]

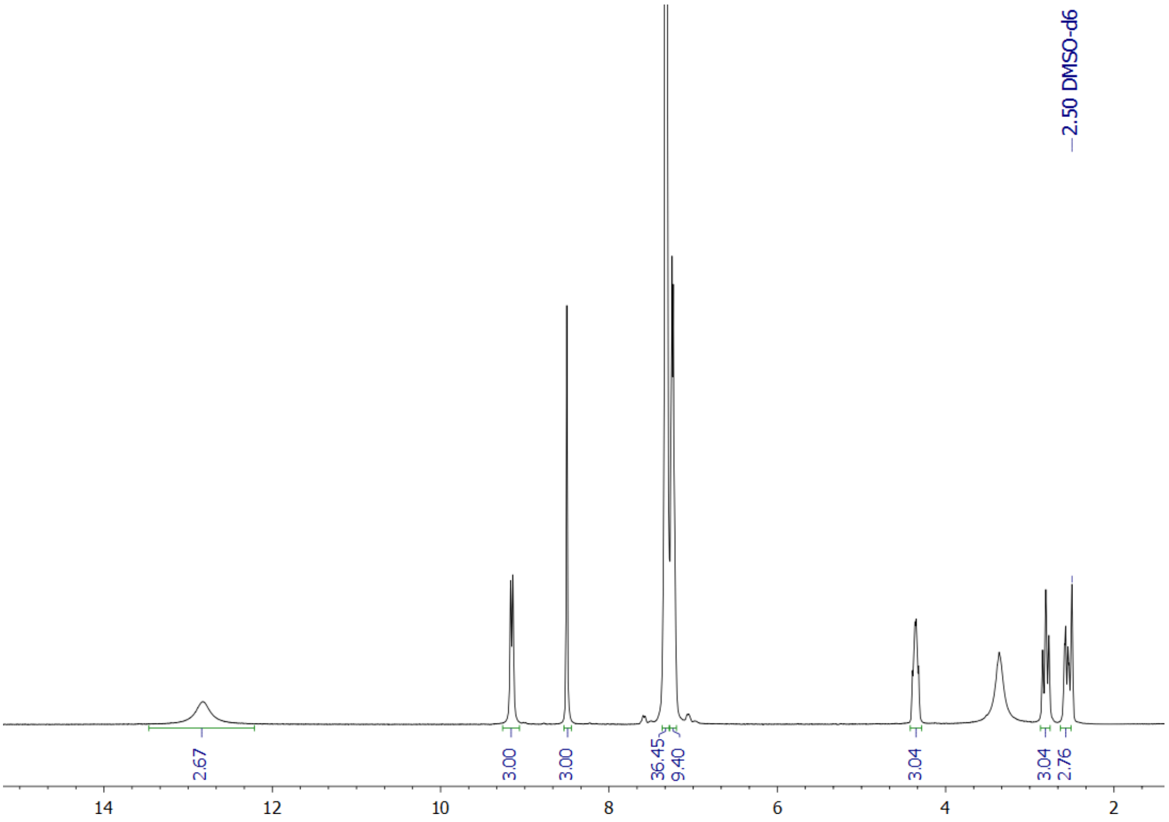

Supplementary Figure 1. ^{1}H NMR (300 MHz DMSO d_6) of L-**1**.

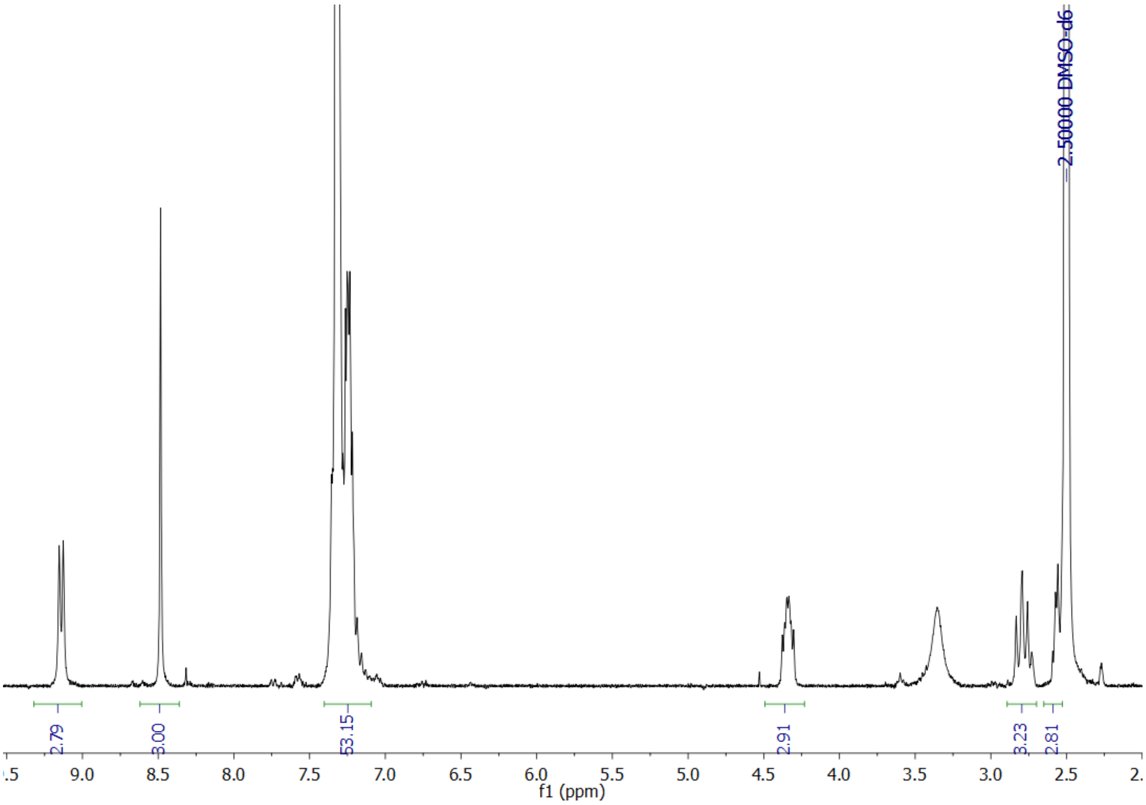

Supplementary Figure 2. ^{1}H NMR (300 MHz DMSO d_6) of D-**1**.

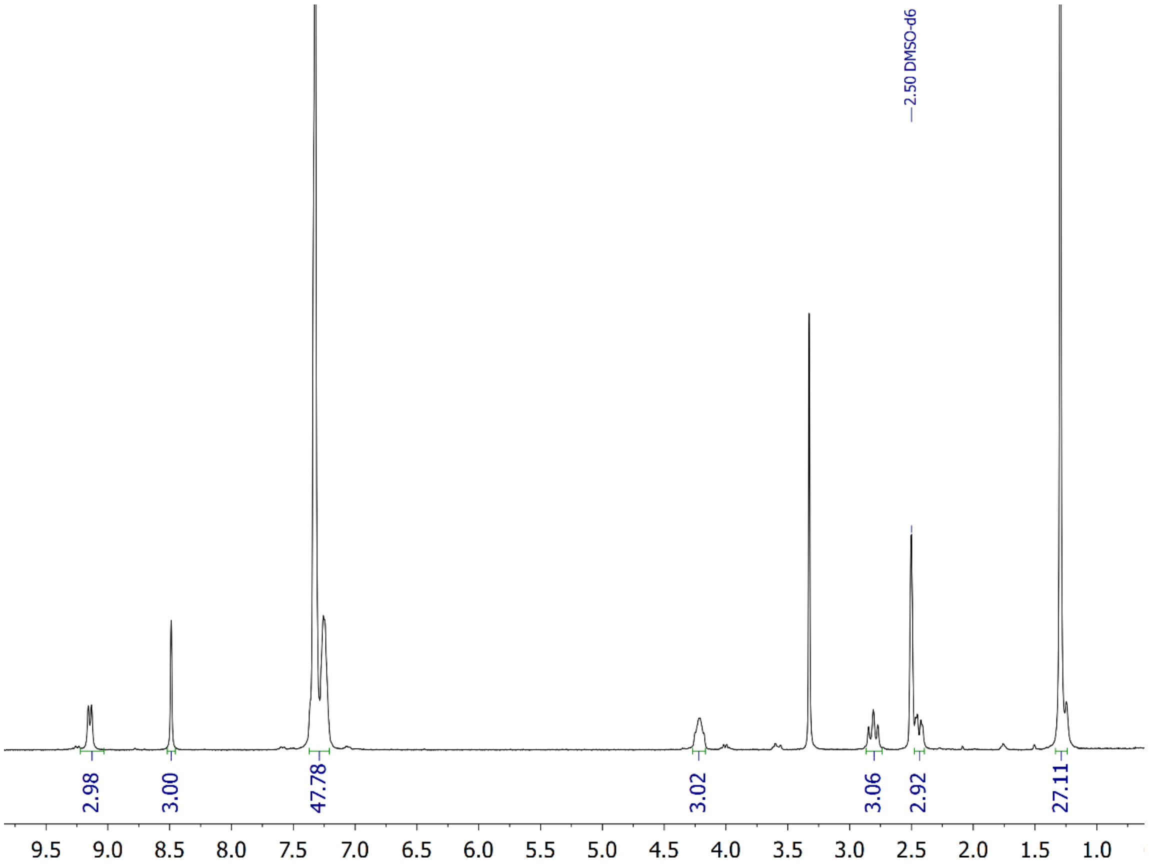

Supplementary Figure 3. ^{1}H NMR (300 MHz DMSO d_6) of L-2.

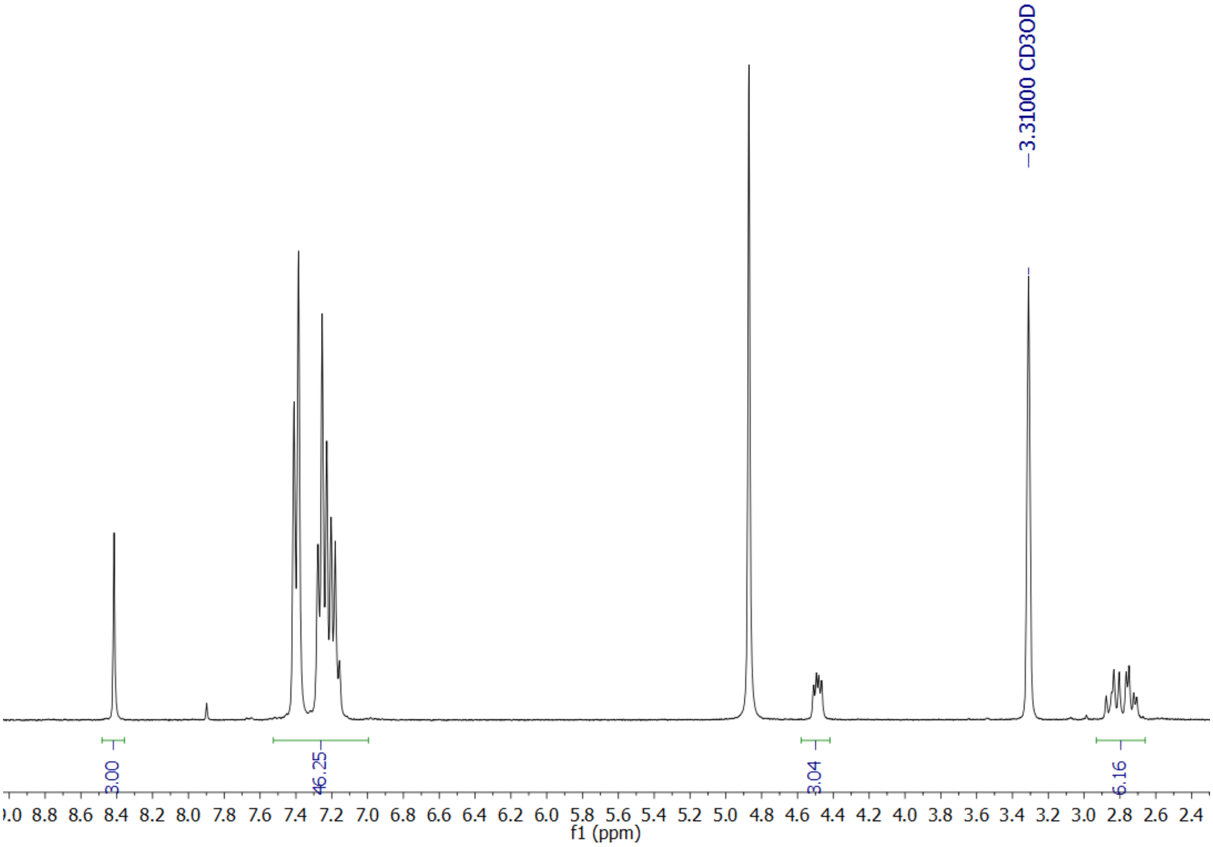

Supplementary Figure 4. ^{1}H NMR (300 MHz MeOD) of L-**1**.

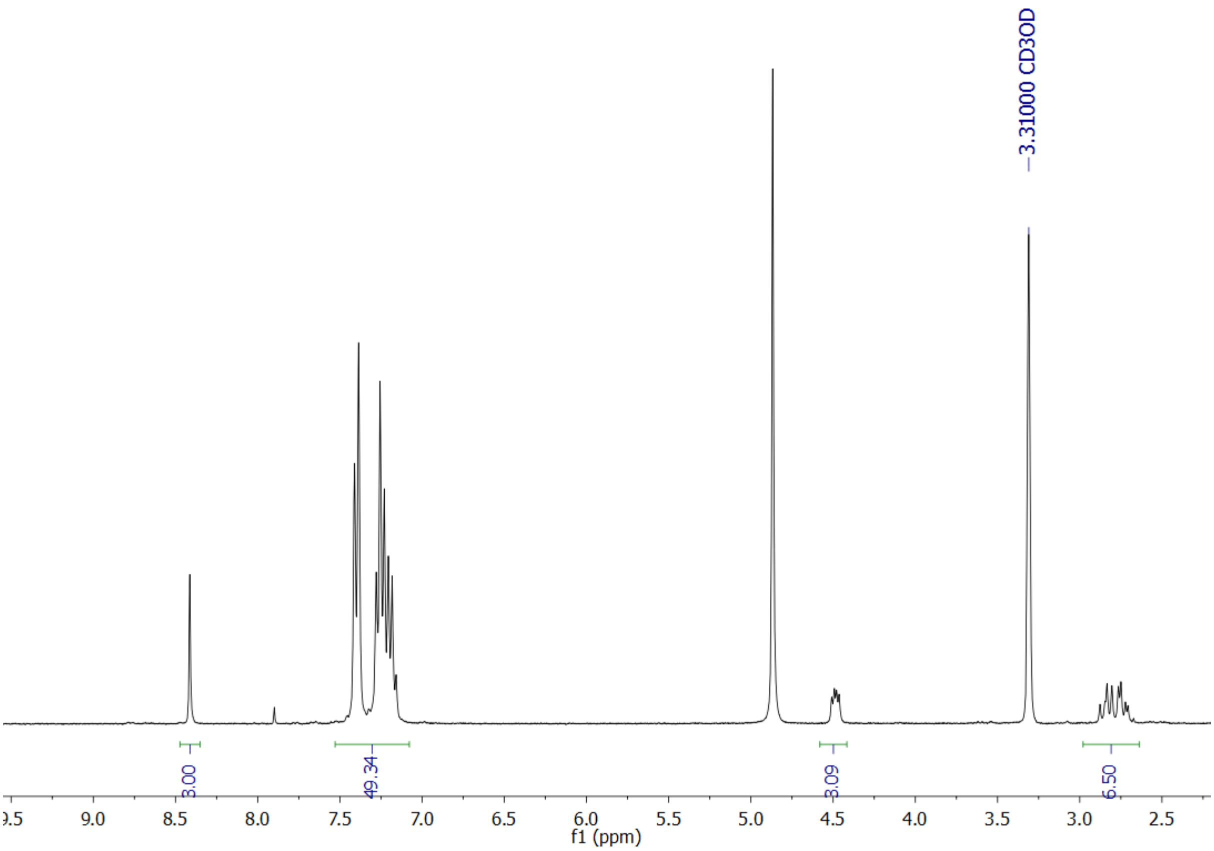

Supplementary Figure 5. ^{1}H NMR (300 MHz MeOD) of D-**1**.

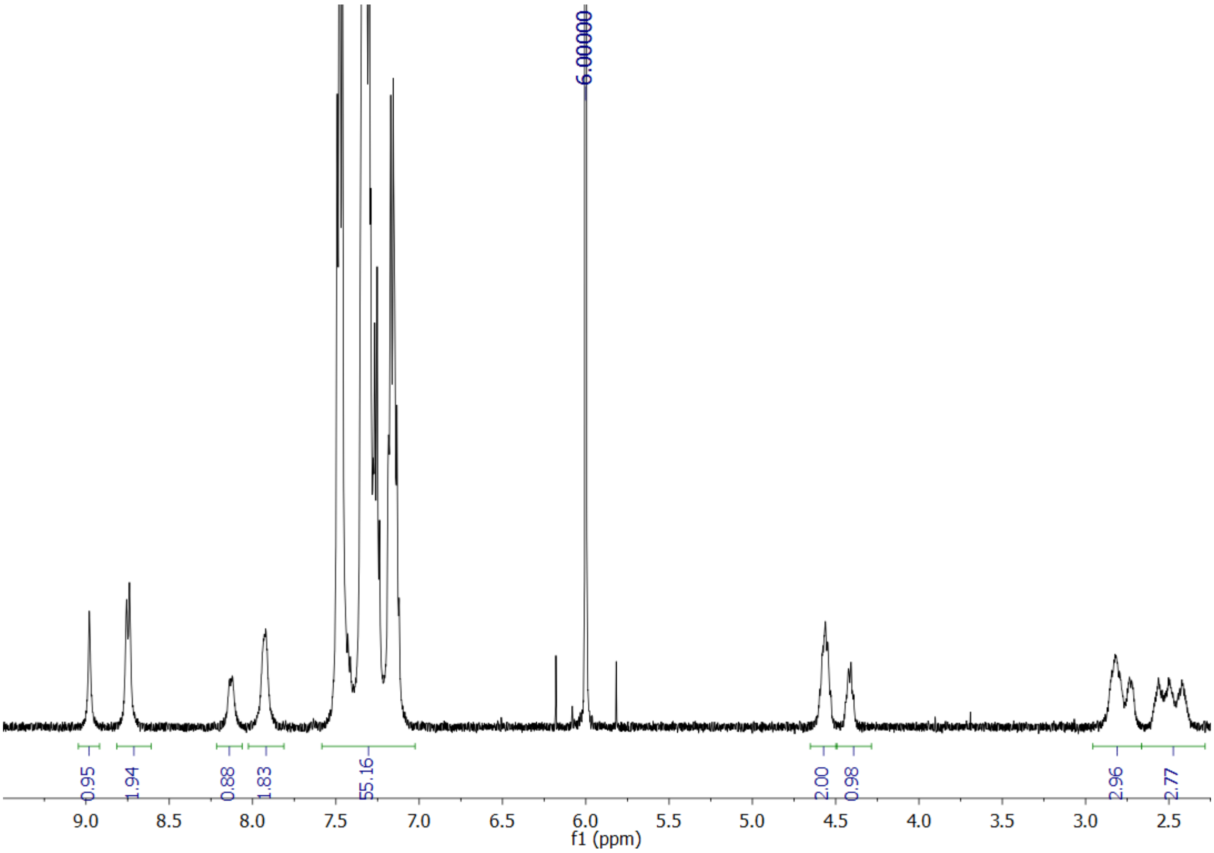

Supplementary Figure 6. ^{1}H NMR (500 MHz TCE d_2) of L-**1**.

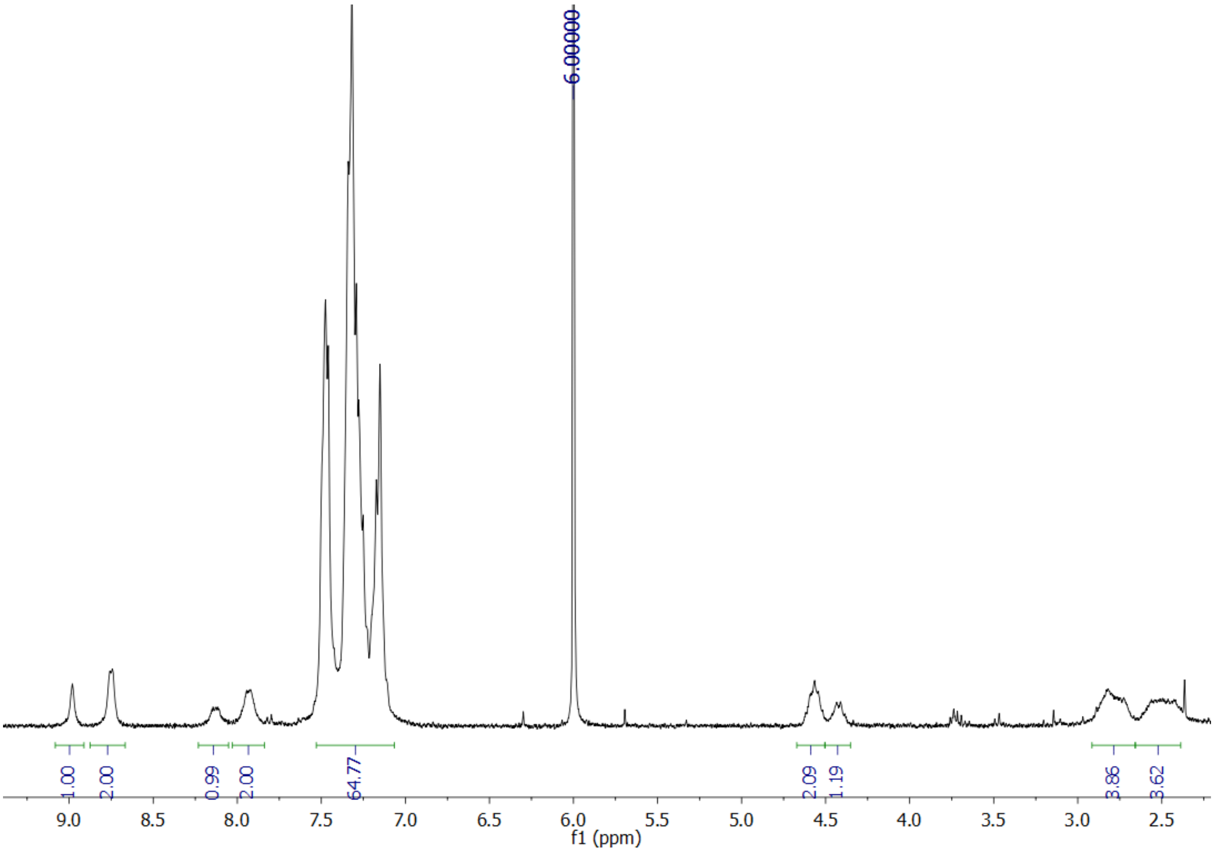

Supplementary Figure 7. ^{1}H NMR (300 MHz TCE d_2) of D-**1**.

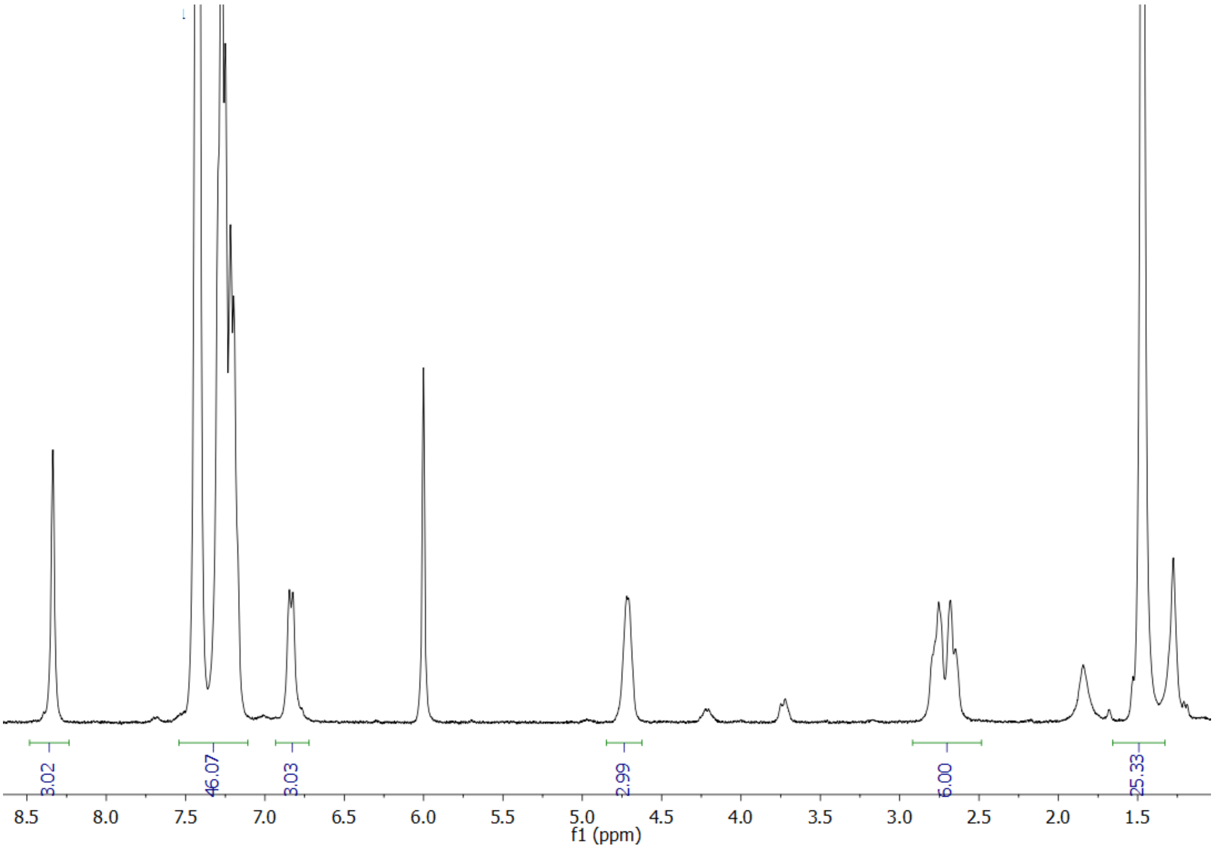

Supplementary Figure 8. ^{1}H NMR (300 MHz TCE d_2) of L-**2**.

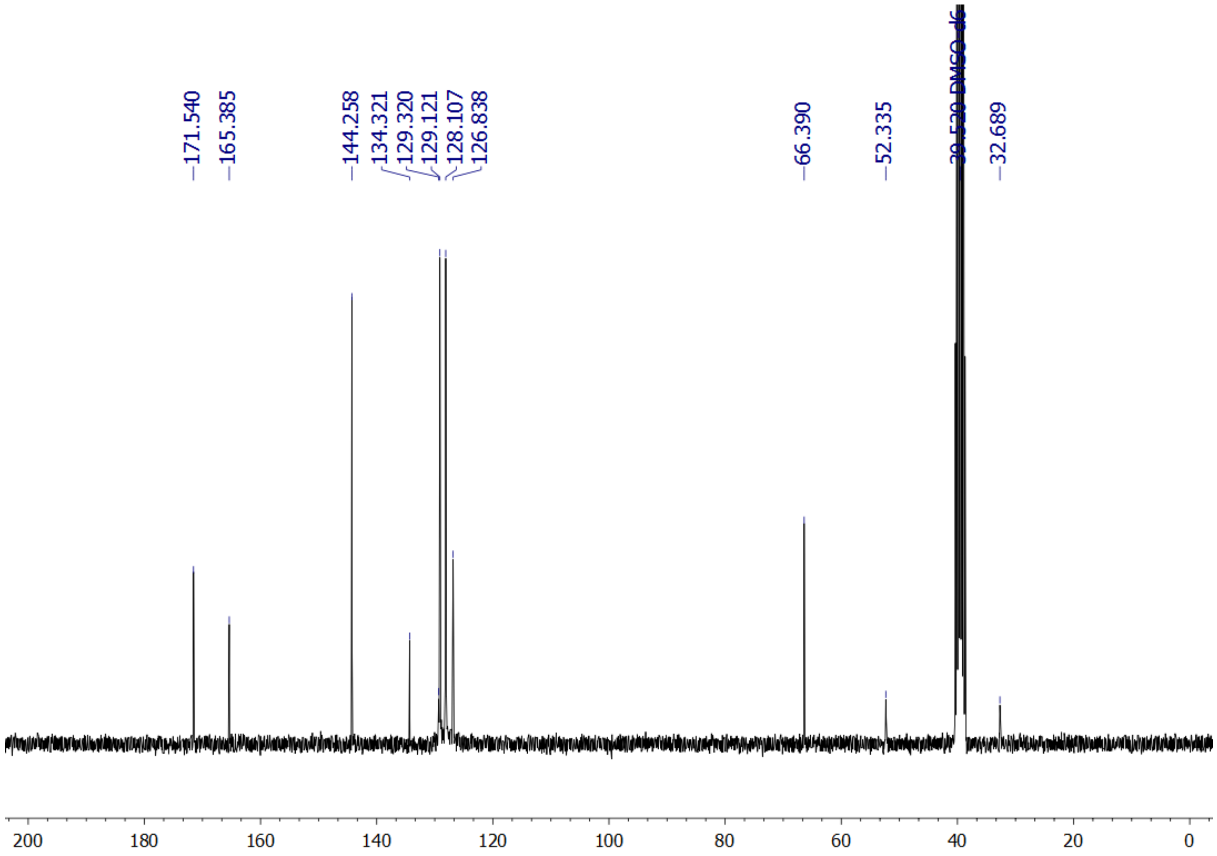

Supplementary Figure 9. ^{13}C NMR (75 MHz DMSO d_6) of L-**1**. (relaxation delay: d1=4.00 s)

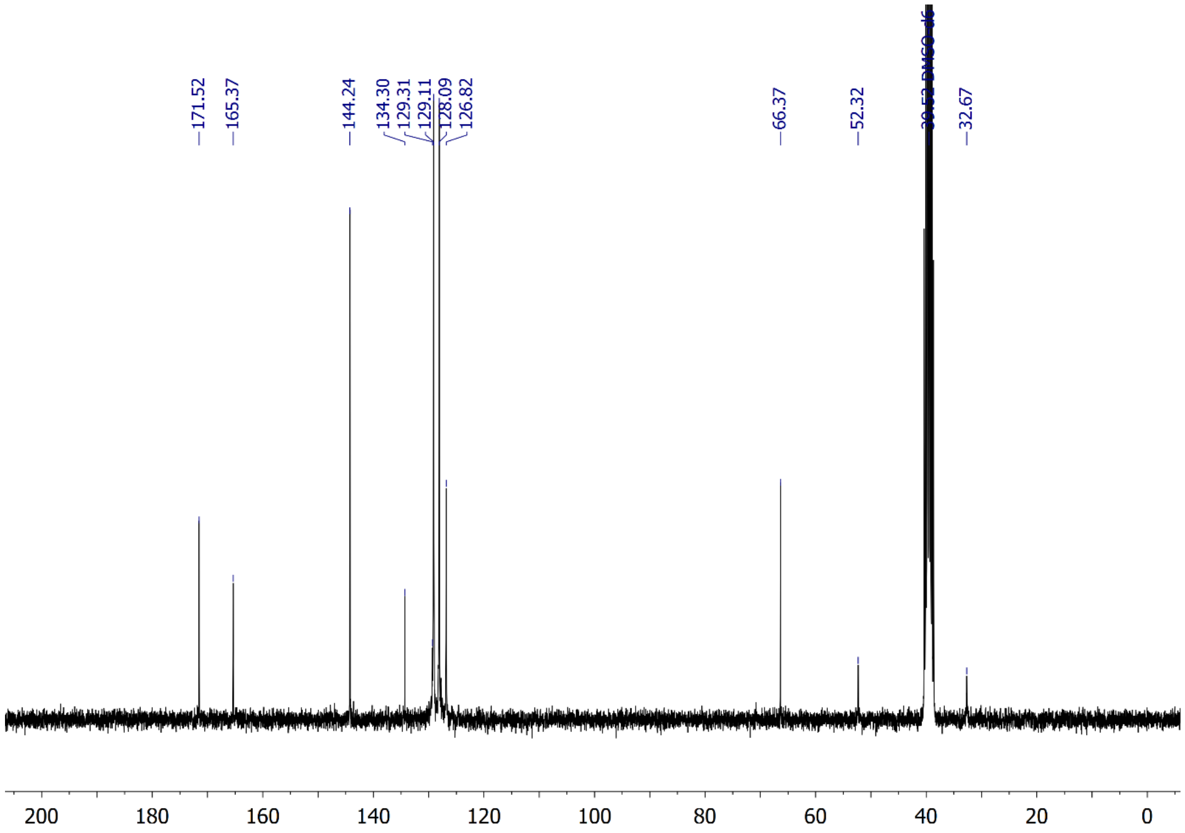

Supplementary Figure 10. ^{13}C NMR (75 MHz DMSO d_6) of D-**1**. (relaxation delay: d1=4.00 s)

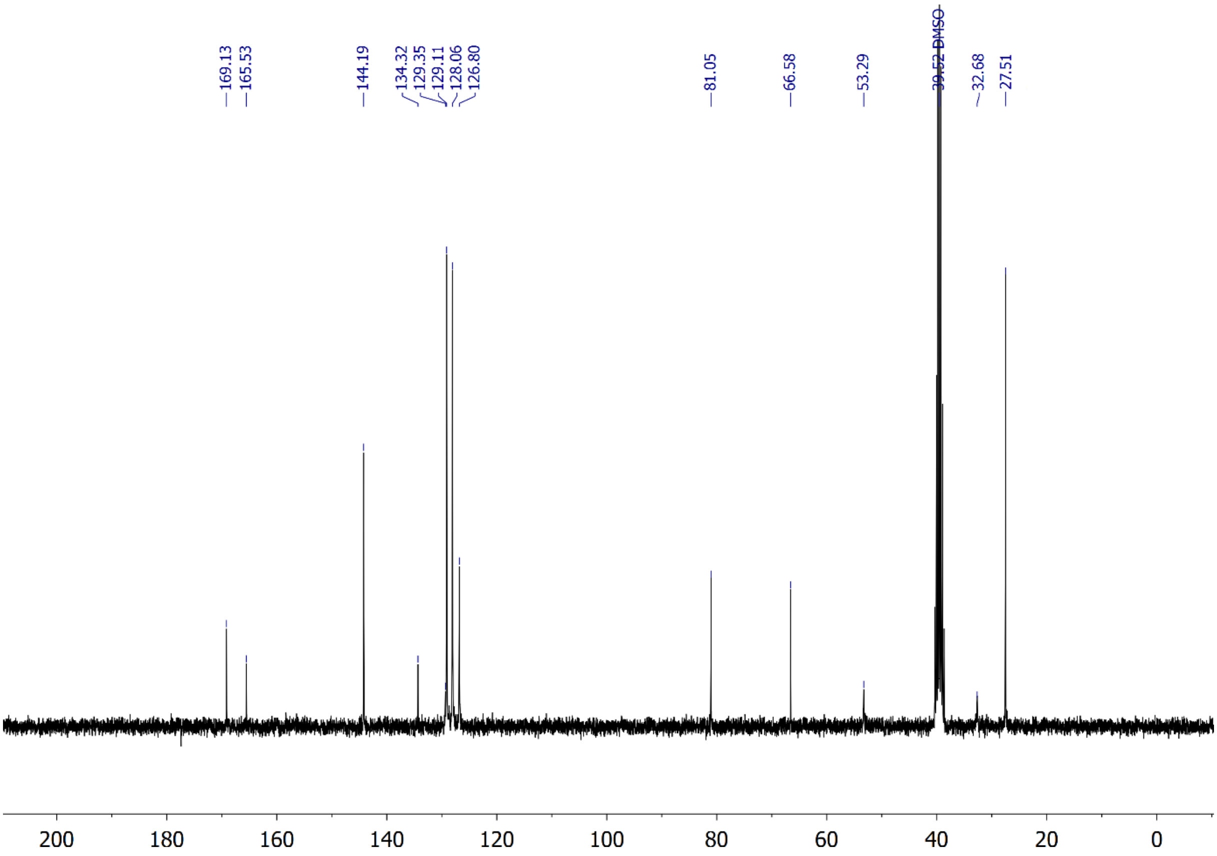

Supplementary Figure 11. ^{13}C NMR (75 MHz DMSO d_6) of L-**2**. (relaxation delay: d1=4.00 s)

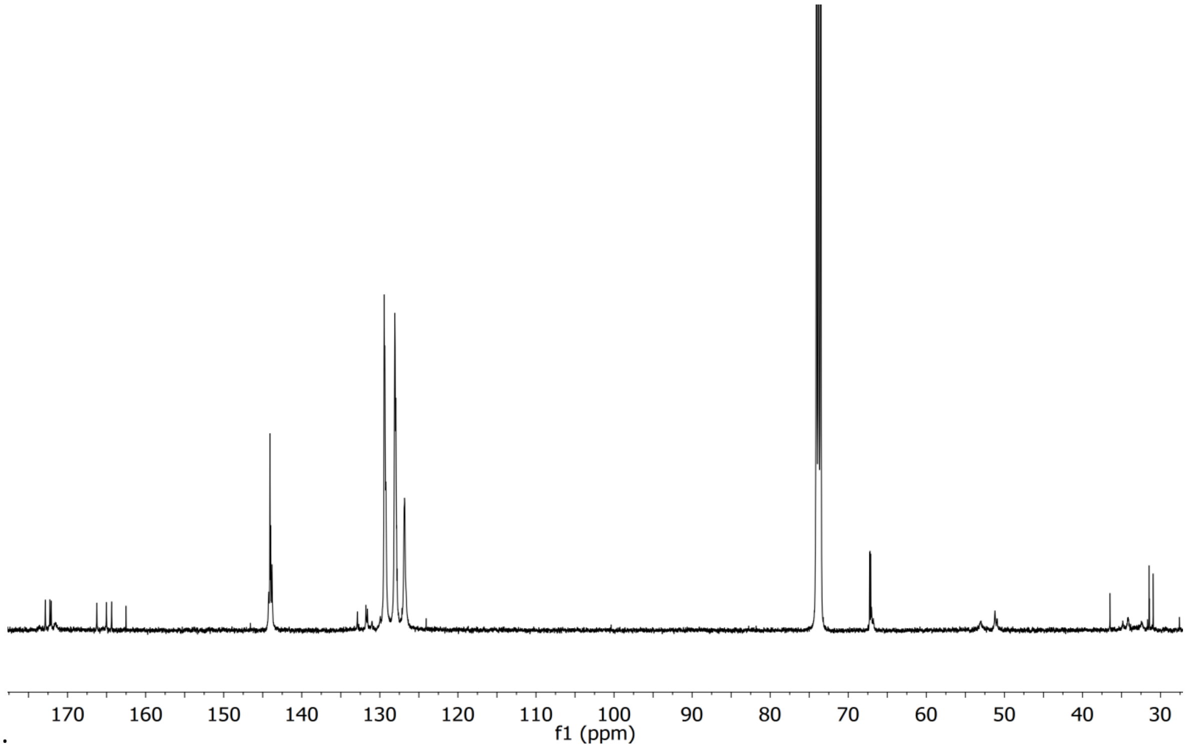

.

Supplementary Figure 12. ^{13}C NMR (100 MHz TCE d_2) of L-**1**. (relaxation delay: d1=4.00 s)

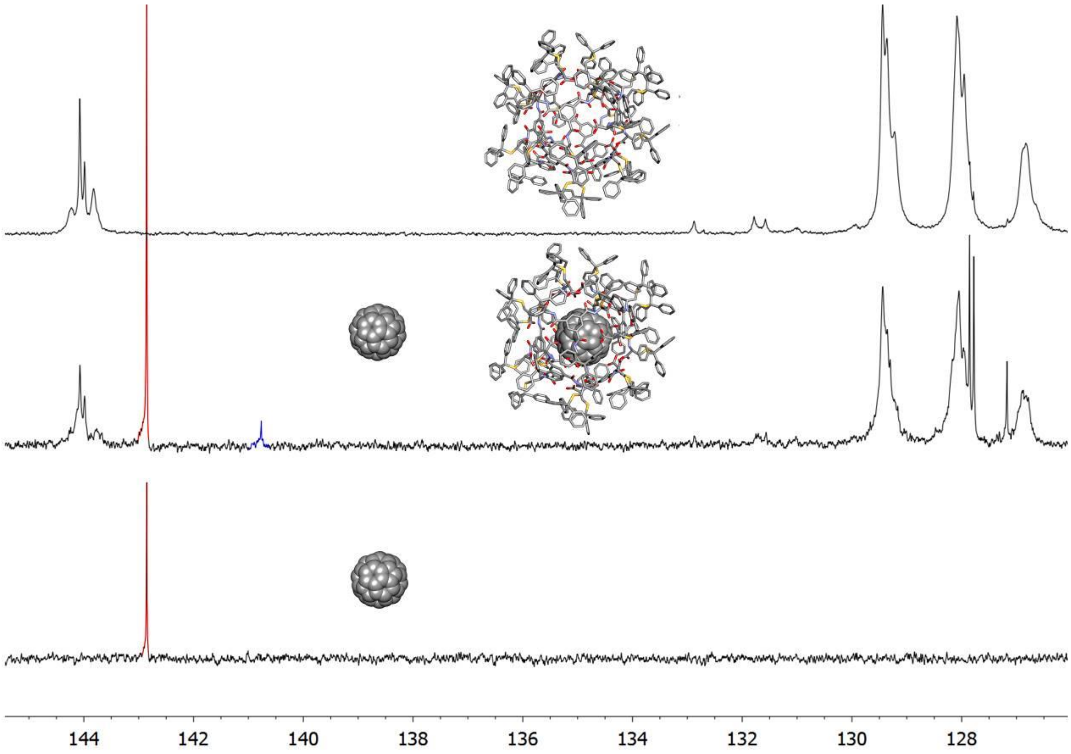

Supplementary Figure 13. ^{13}C NMR (100 MHz TCE d_2) stacked spectra of free capsule, L-**1** capsule with C_{60} fullerene, and free C_{60} fullerene. Red signals: free fullerene. Blue signal: encapsulated fullerene.

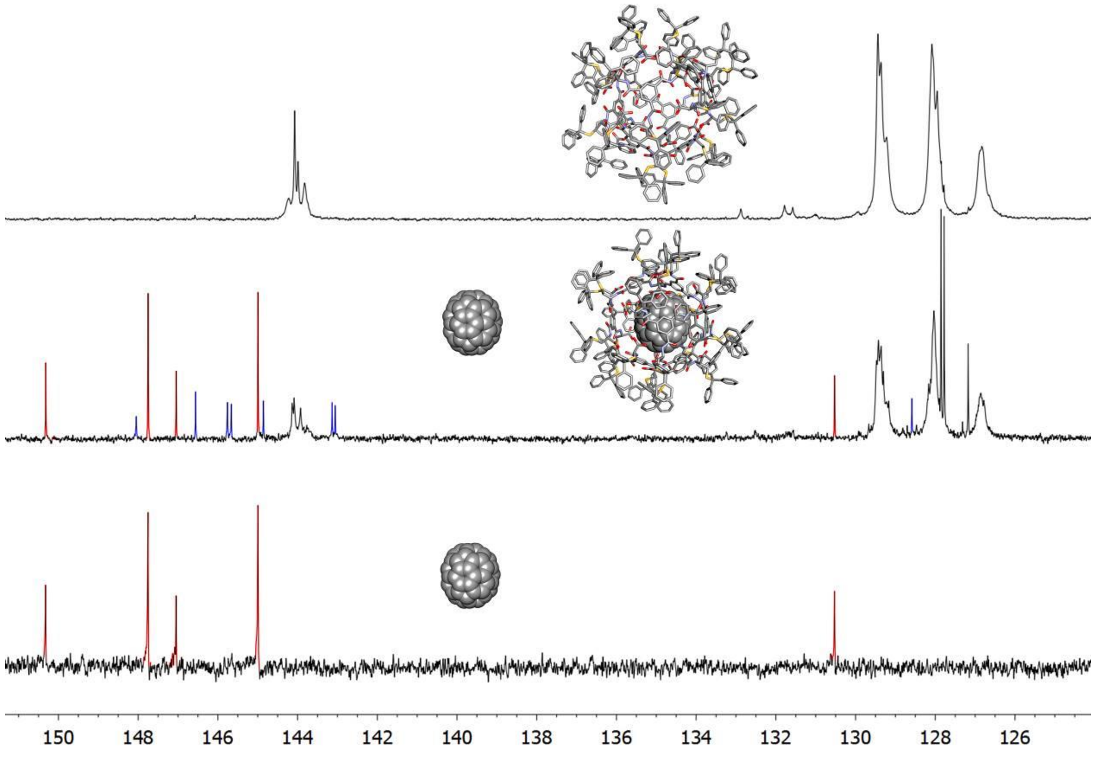

Supplementary Figure 14. ^{13}C NMR (150 MHz TCE d_2) stacked spectra of free capsule, L-**1** capsule with C_{70} fullerene, and free C_{70} fullerene. Red signals: free fullerene. Blue signals: encapsulated fullerene.

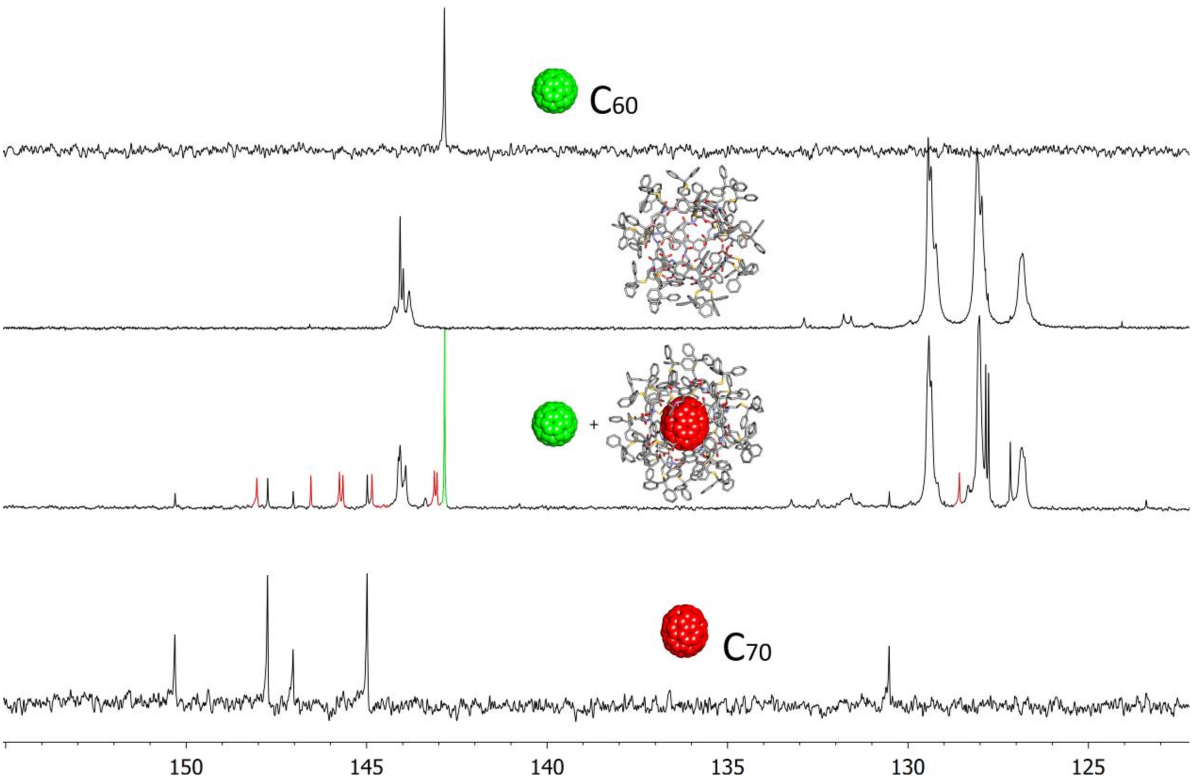

Supplementary Figure 15. ^{13}C NMR (150 MHz TCE d_2) stacked spectra of free C_{60} fullerene, free capsule, capsule with C_{60} and C_{70} fullerene in 1:1:1 molar ratio, and free C_{70} fullerene.

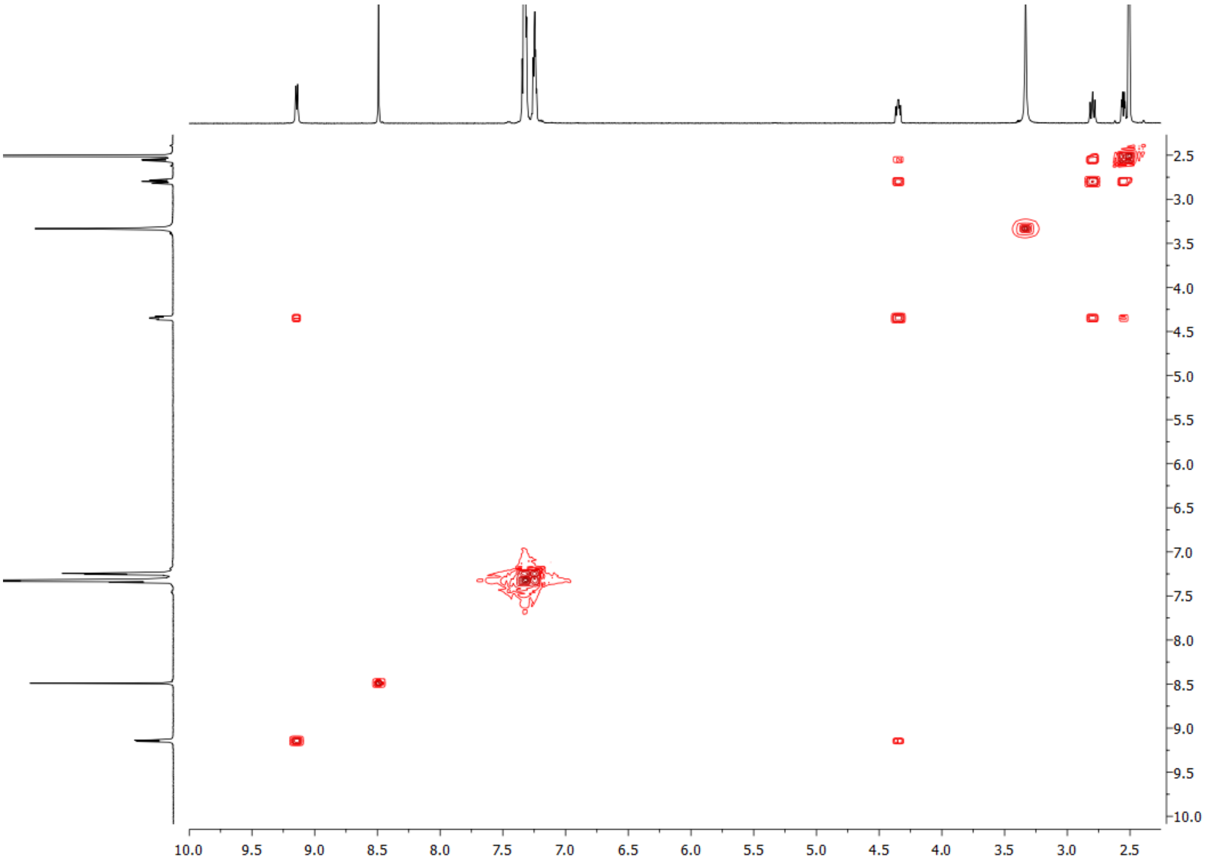

Supplementary Figure 16. COSY NMR (600 MHz DMSO d_6) of L-1.

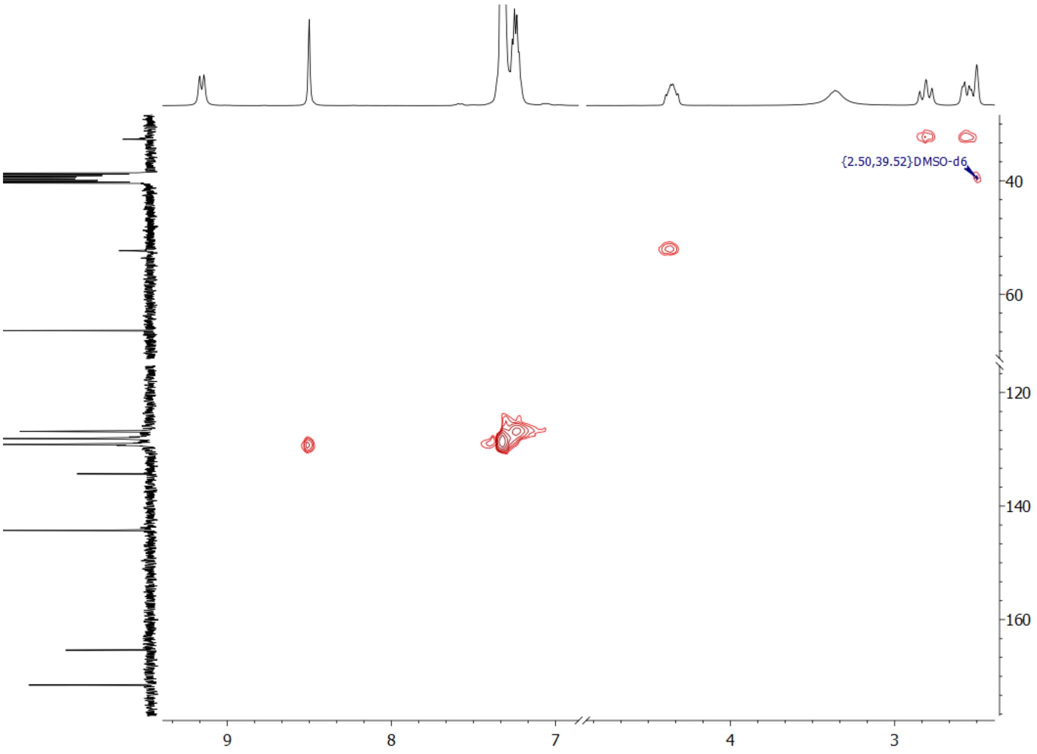

Supplementary Figure 17. ^{1}H-^{13}C HSQC NMR (300/75 MHz DMSO d_6) of L-**1**. Empty regions removed for clarity.

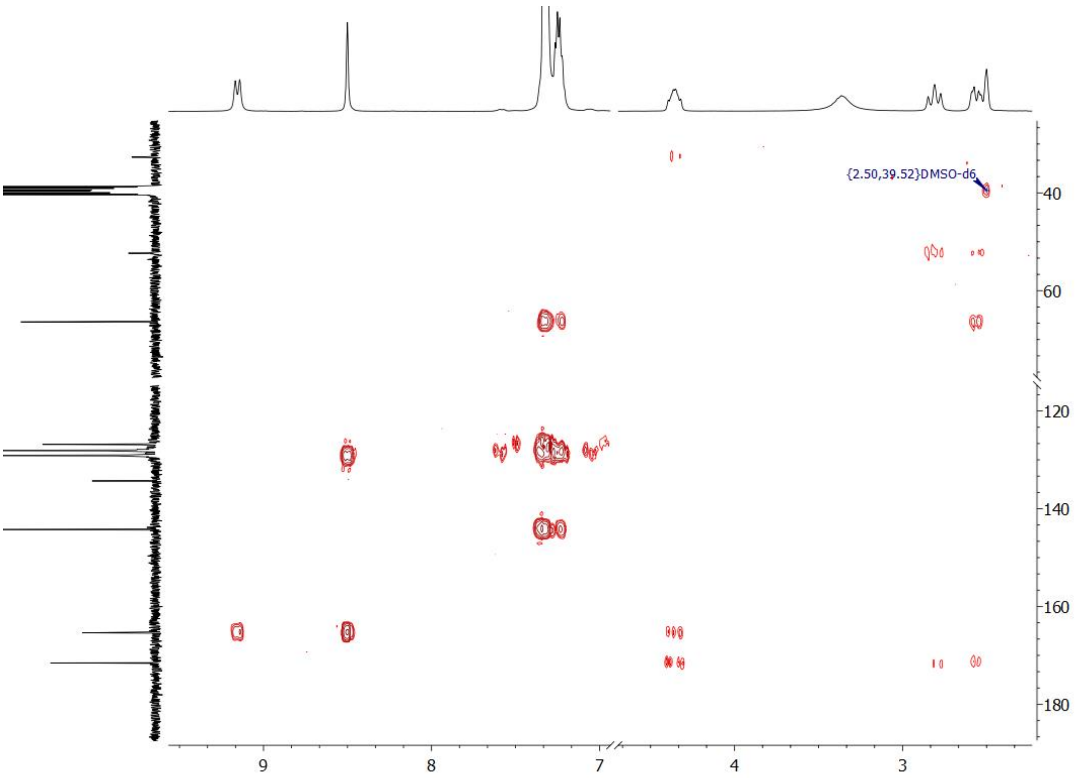

Supplementary Figure 18. ^{1}H-^{13}C HMBC NMR (300/75 MHz DMSO d_6) of L-**1**. Empty regions removed for clarity.

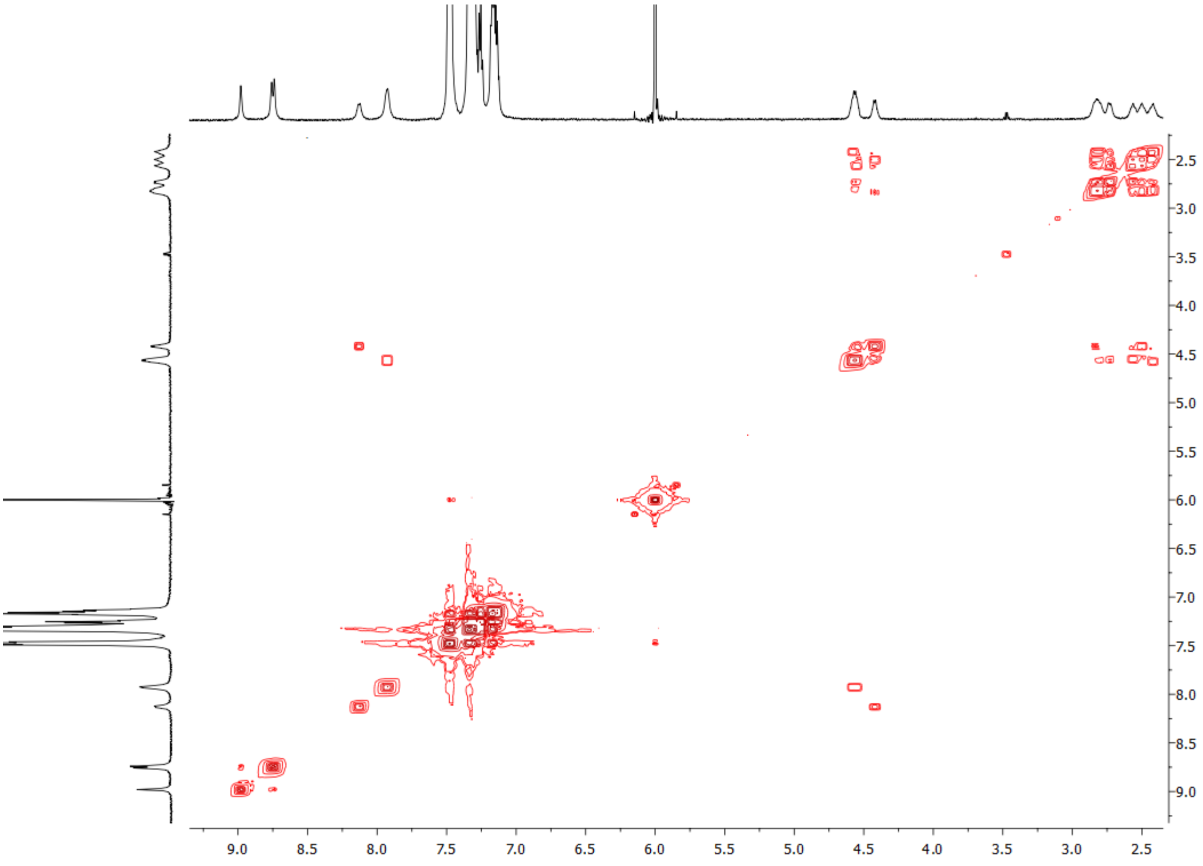

Supplementary Figure 19. COSY NMR (600 MHz TCE d_2) of L-1

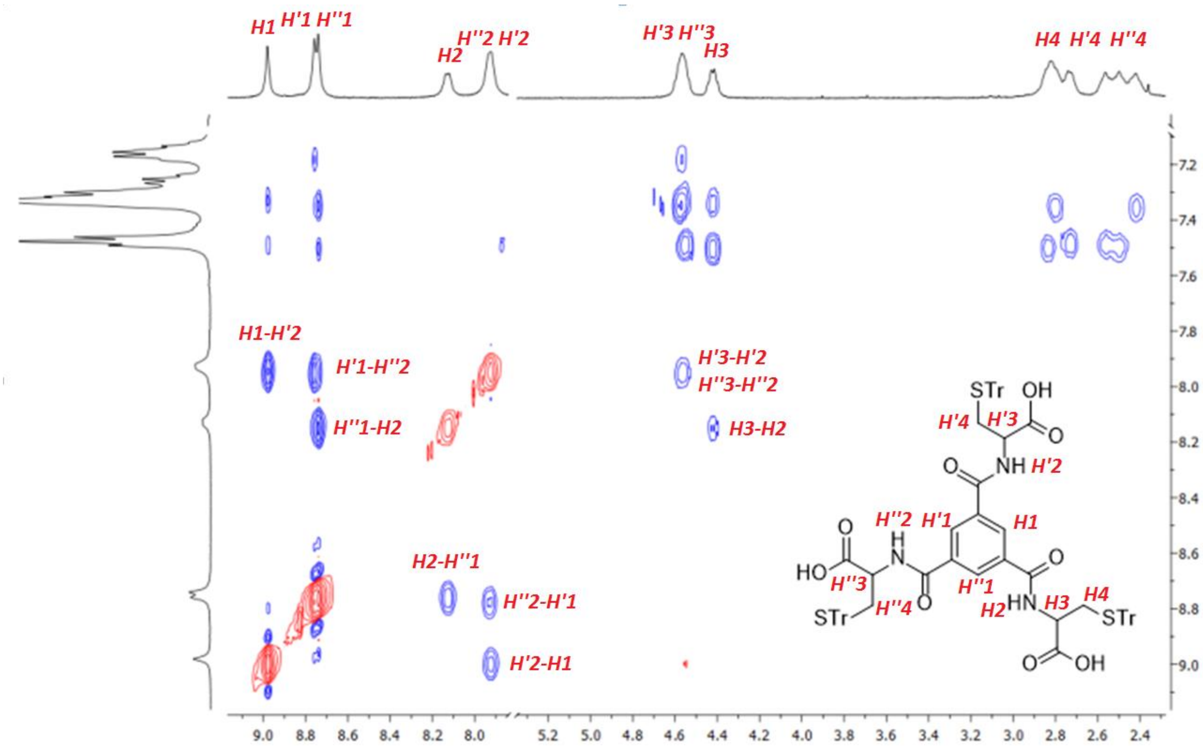

Supplementary Figure 20. EASY-ROESY NMR (500 MHz TCE d_2) of L-1 with assigned ROE couplings *(p15=250 msec).* Empty regions removed for clarity.

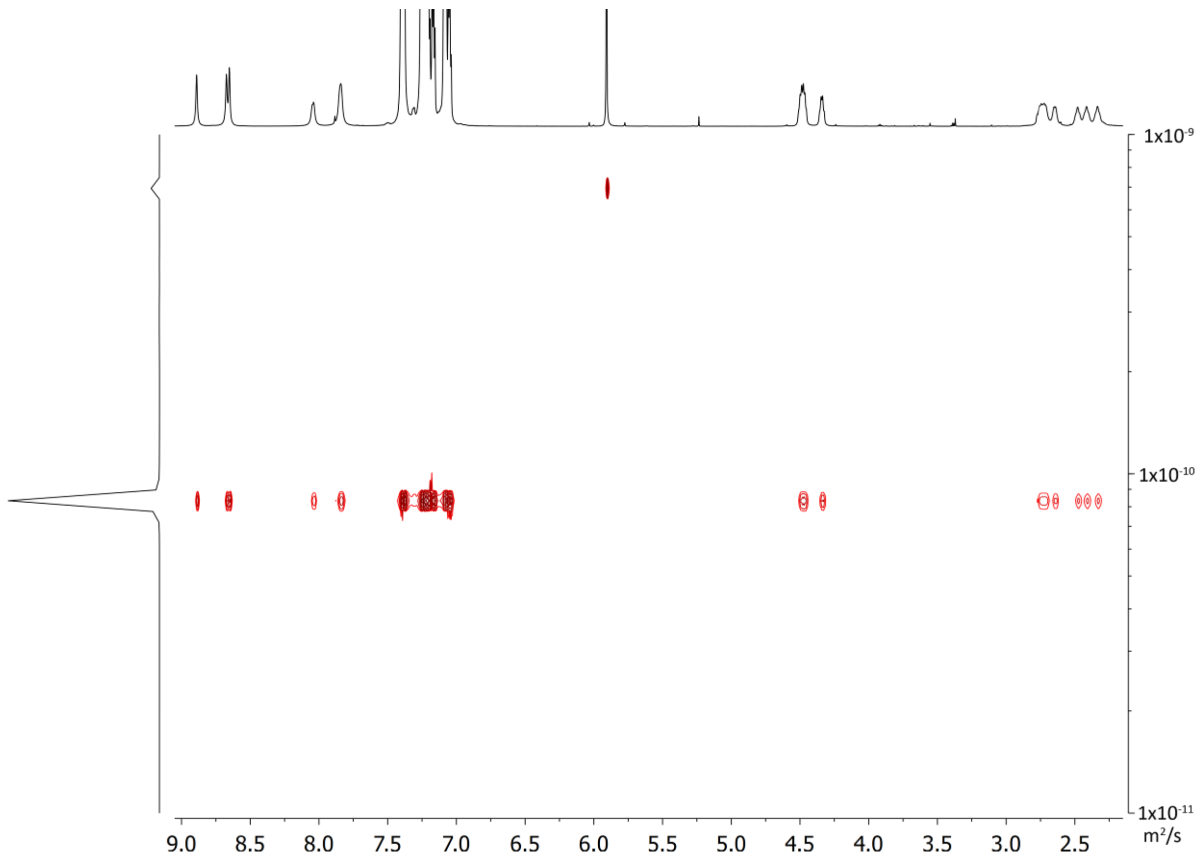

Supplementary Figure 21. ^{1}H (700 MHz TCE d_2) DOSY spectrum of L-1.

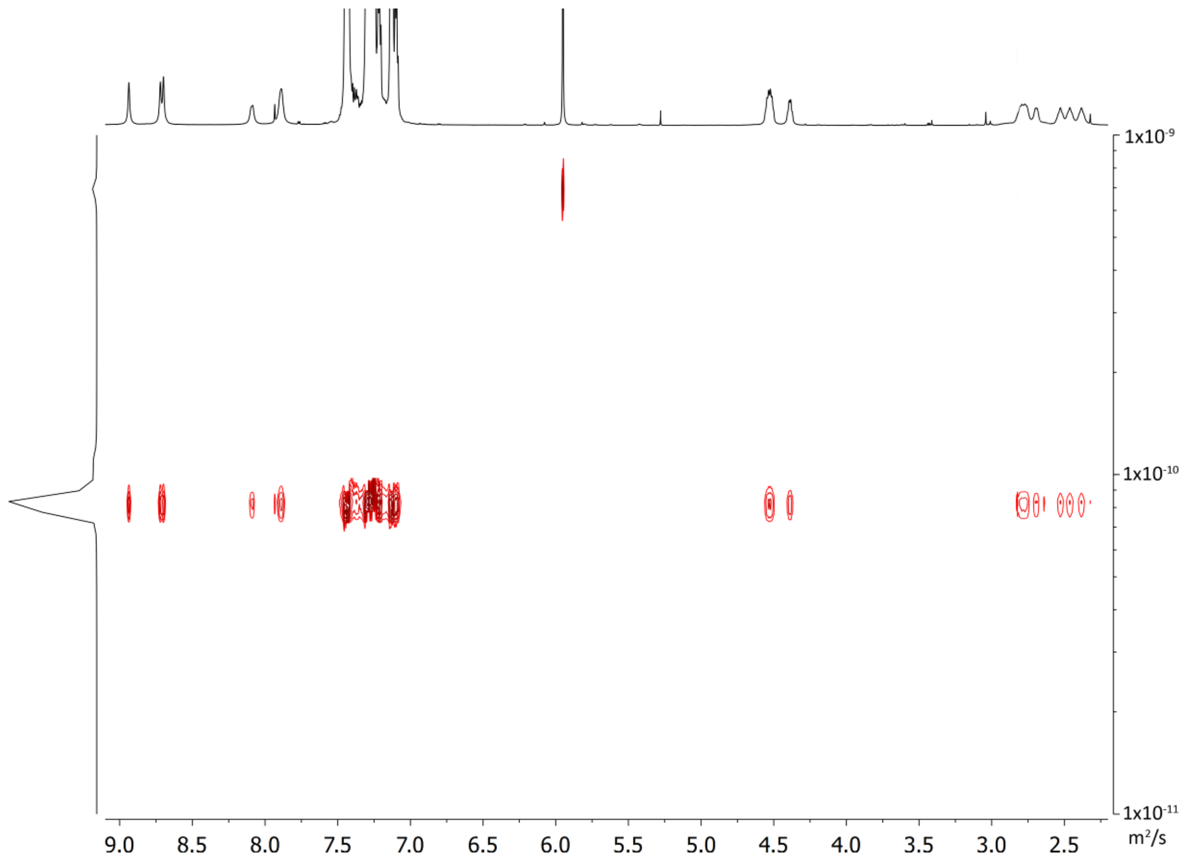

Supplementary Figure 22. ^{1}H (700 MHz TCE d_2) DOSY spectrum of D-**1**.

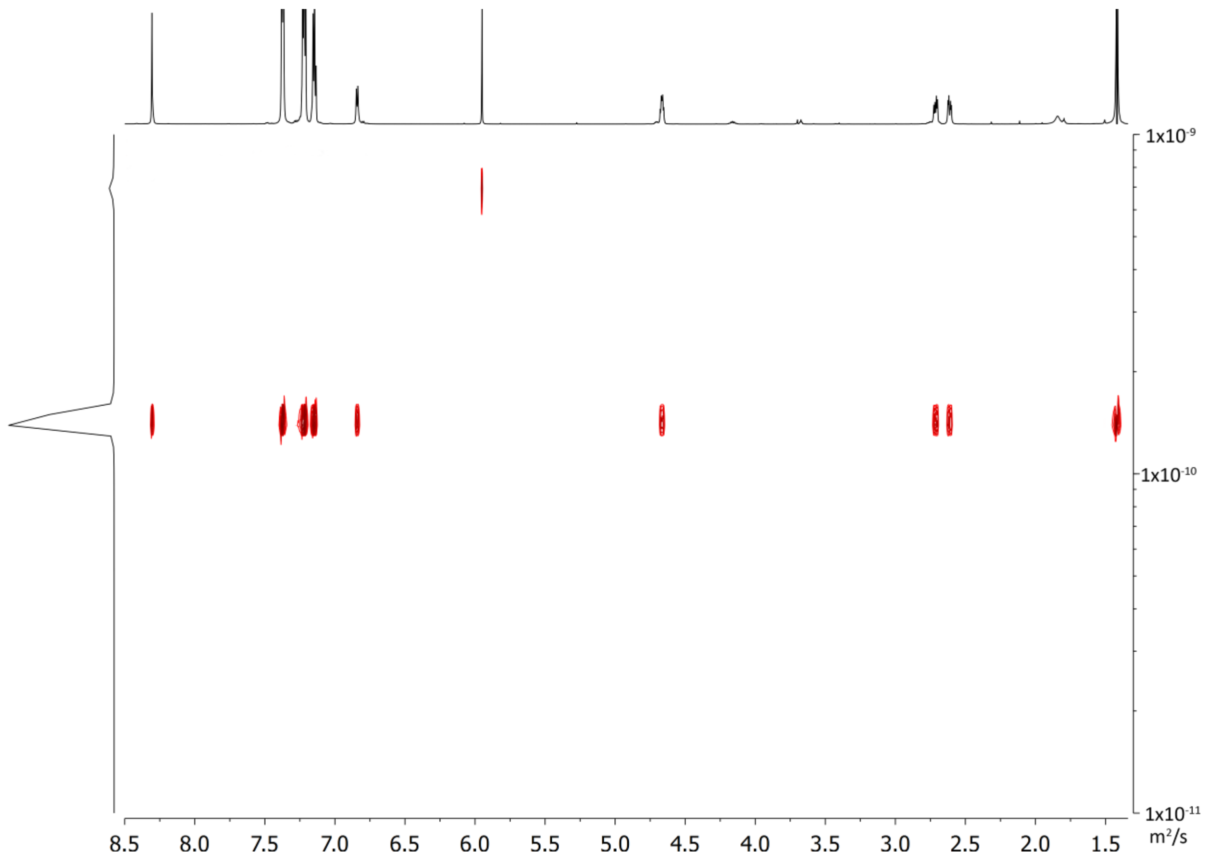

Supplementary Figure 23. ^{1}H (700 MHz TCE d_2) DOSY spectrum of L-**2**.

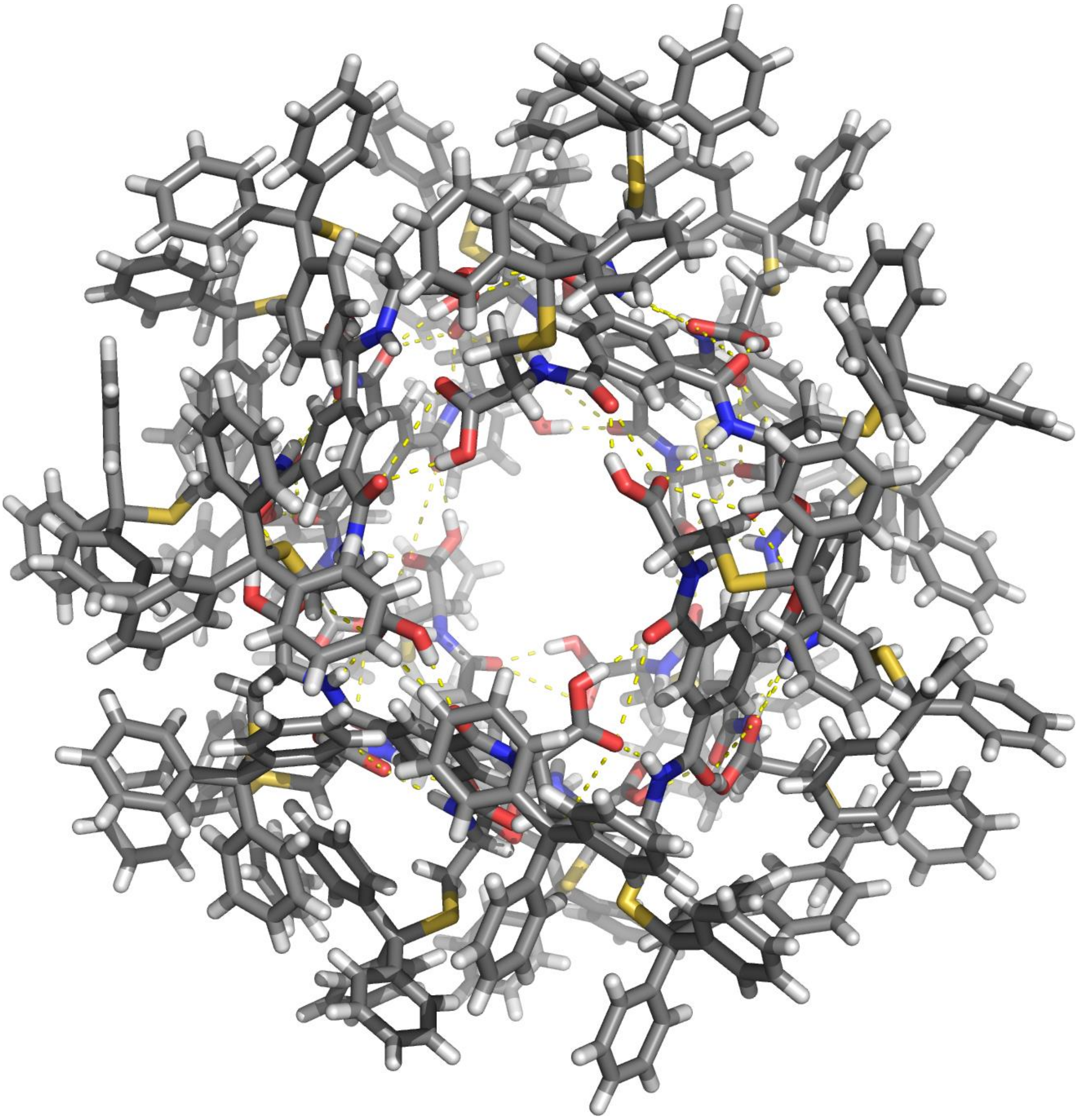

Supplementary Figure 24. The nanocapsule in the solid state; Red = O, yellow = S, blue = N. Disorder has been removed for clarity.

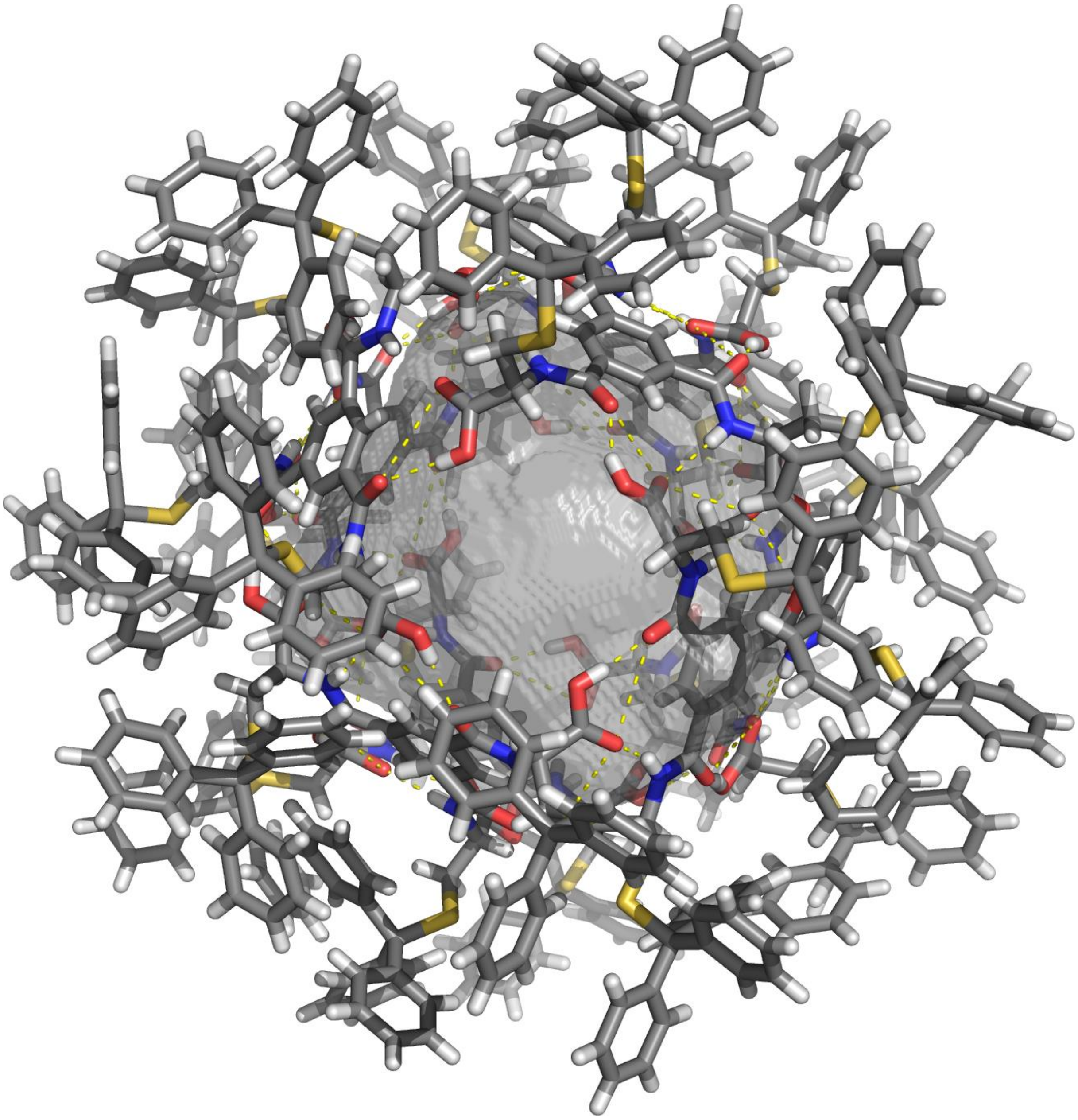

Supplementary Figure 25. The nanocapsule in the solid state; Red = O, yellow = S, blue = N. Disorder has been removed for clarity. The encapsulated void volume of 1.7 nm^3 is shown as 50% transparent gray isosurface.

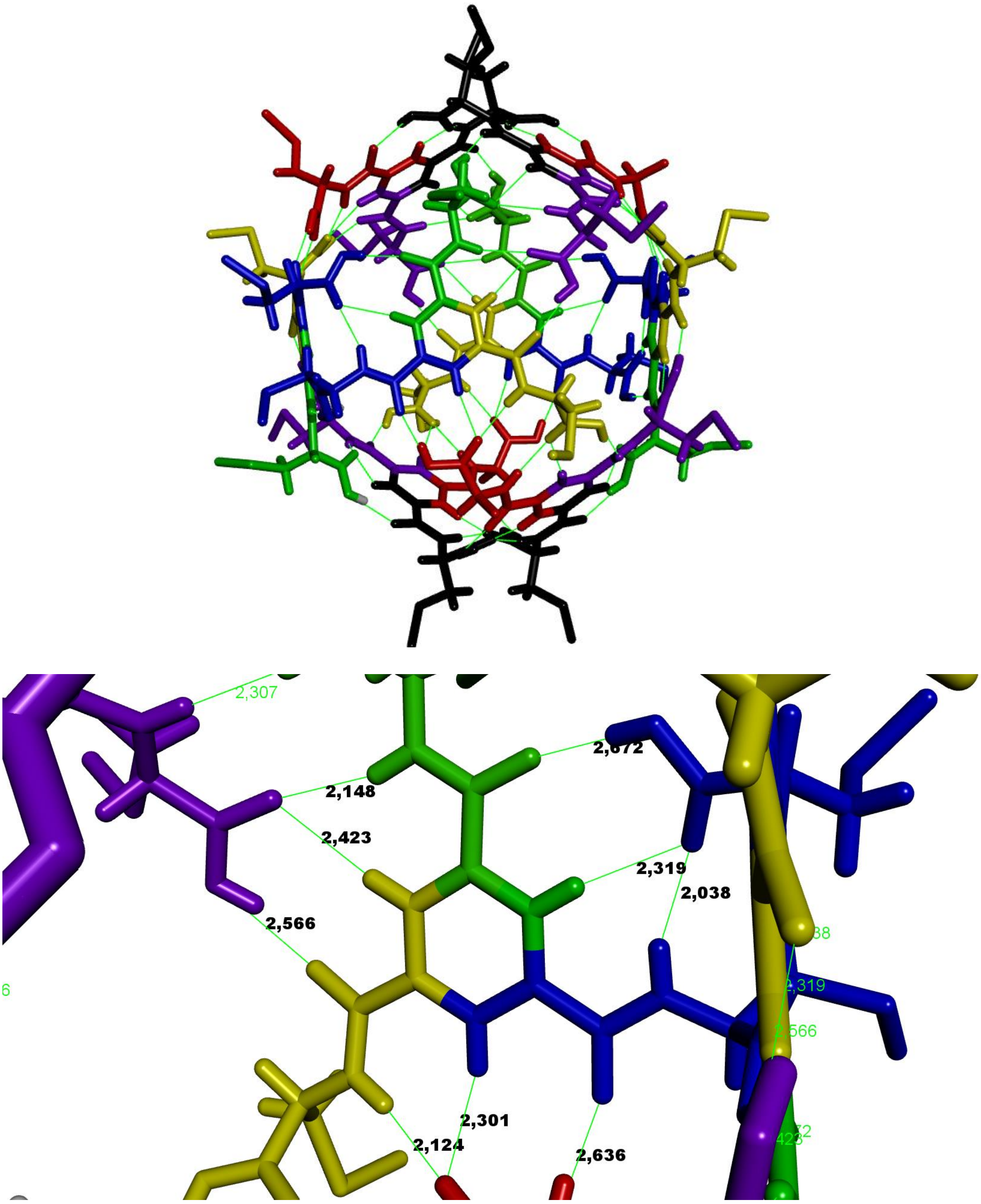

Supplementary Figure 26. Desymetrisation of the tripod arms in the capsule. Top: Full capsule in color form (Trityl groups removed for clarity). Bottom: Part of the capsule with selected distances.

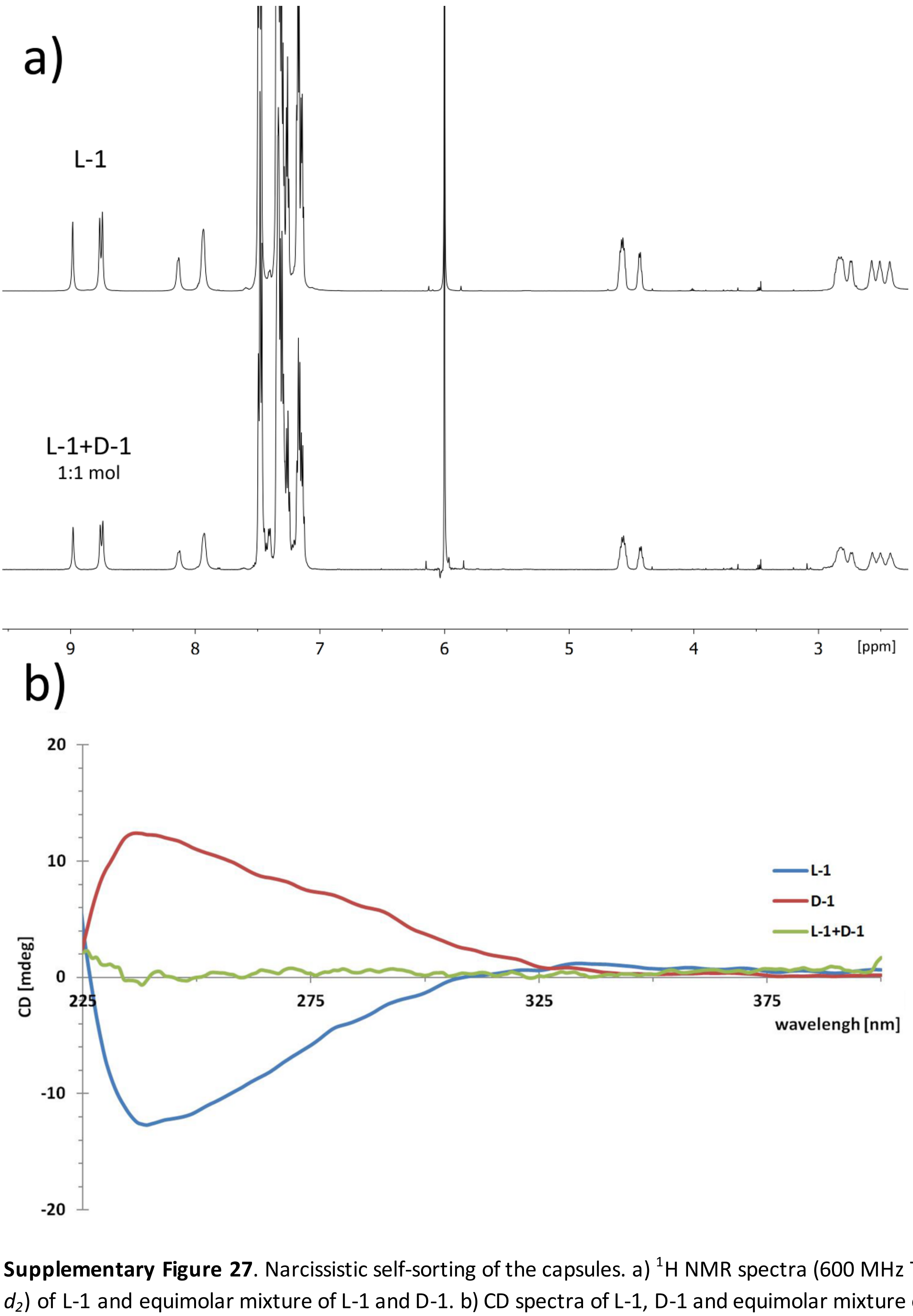

Supplementary Figure 27. Narcissistic self-sorting of the capsules. a) ^{1}H NMR spectra (600 MHz TCE-d_2) of L-1 and equimolar mixture of L-1 and D-1. b) CD spectra of L-1, D-1 and equimolar mixture of L-1 and D-1 recorded in dichloroethane at C≈5x10^{-4} M and d=1mm.

a)

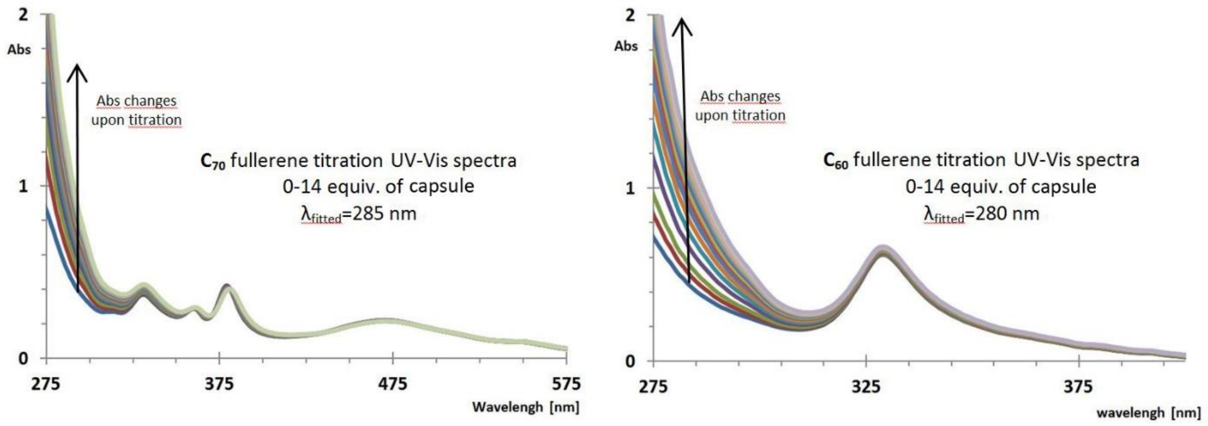

b)

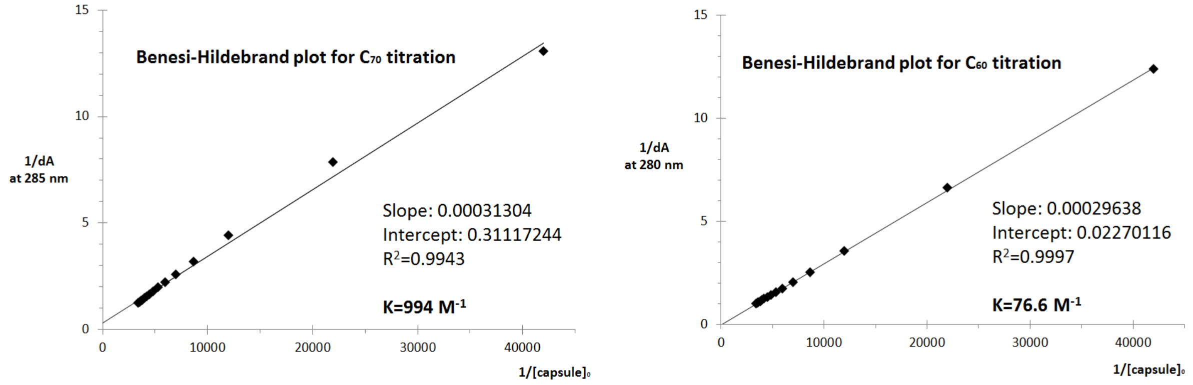

c)

	C70			C60	
C_o	conversion %	K	C_o	Conversion %	K
1,80E-03	50%	**1111,11**	1,80E-03	10%	**68,59**
	50%			90%	

Supplementary Figure 28. Binding constant determination. a) UV-Vis spectra obtained during fullerene titration (C_{70} and C_{60} respectively). b) Benesi-Hildebrand plots with regression lines and K values. c) Binding constant determination from ^{13}C NMR spectra.

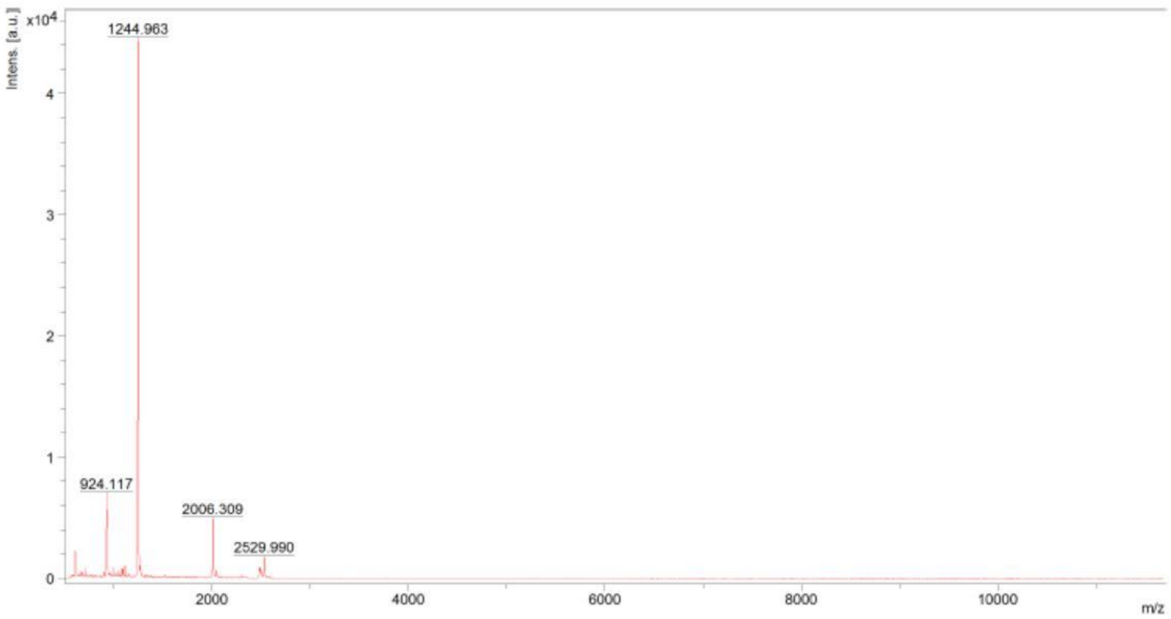

Supplementary Figure 29. MALDI MS TOF/TOF spectrum of L-1 in negative ion mode (DCTB matrix). Calc. $[M\text{-}H]^-$= 1244.3653 m/z. Found: $[M\text{-}H]^-$ 1244.963, $[2M\text{-}2H\text{+}K]^-$ 2529.990 m/z.

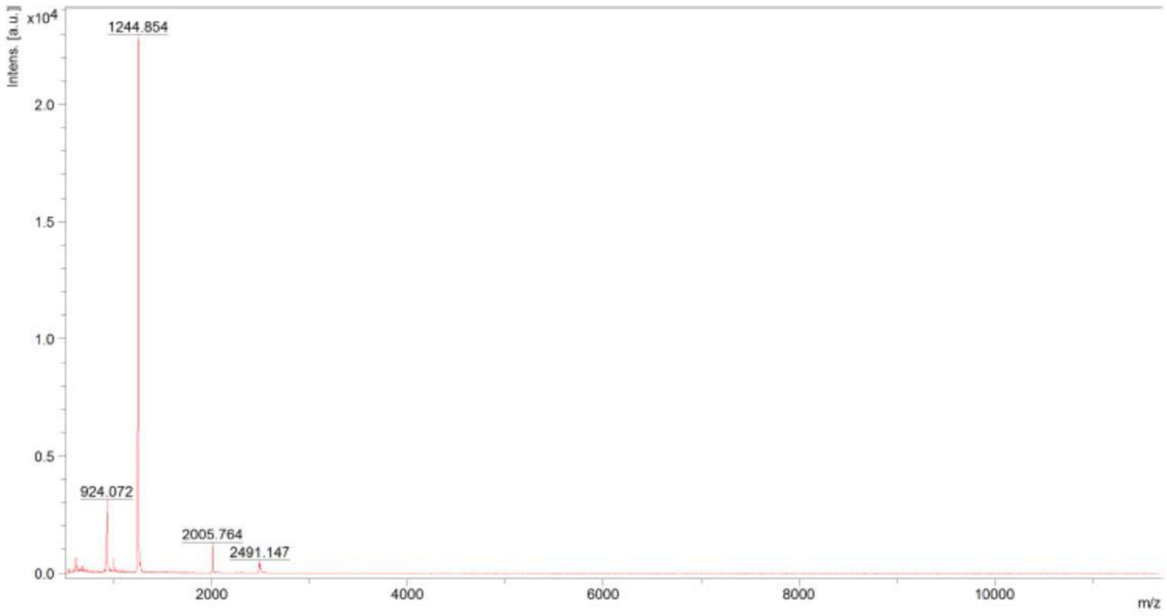

Supplementary Figure 30. MALDI MS TOF/TOF spectrum of D-1 in negative ion mode (DCTB matrix). Calc. $[M\text{-}H]^-$= 1244.3653 m/z. Found: $[M\text{-}H]^-$ 1244.963, $[2M\text{-}H]^-$ 2491.147 m/z.

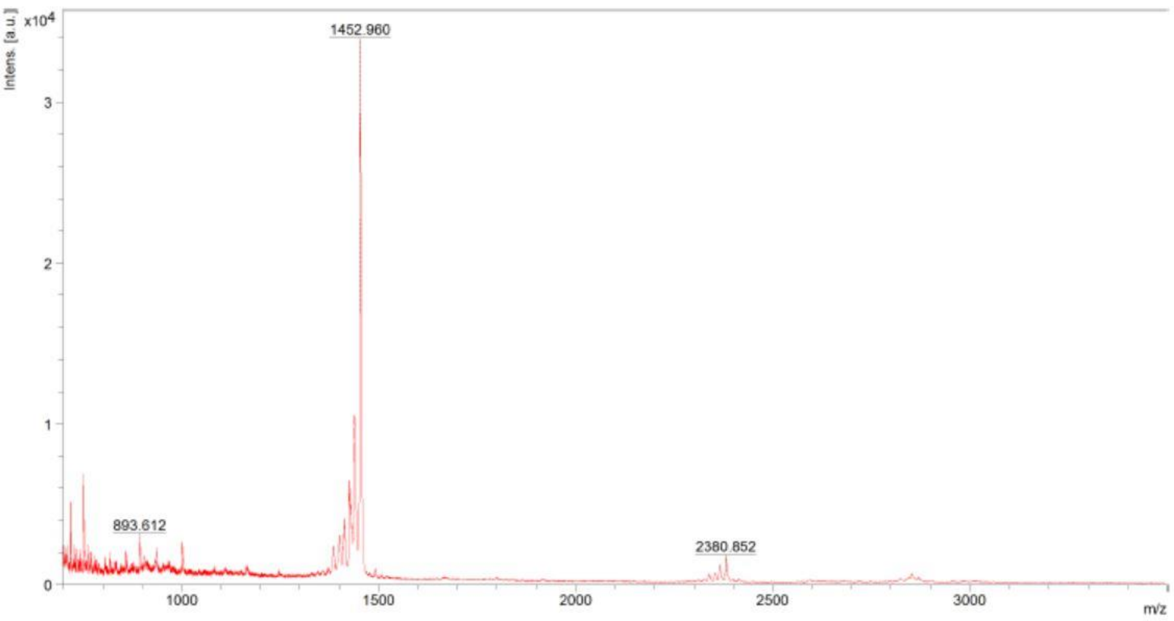

Supplementary Figure 31. MALDI MS TOF/TOF spectrum of L-**2** in positive ion mode (DCTB matrix). Calc. $[M+K]^+$= 1452.5237 m/z. Found: $[M+K]^+$= 1452.960 m/z.

Supplementary Table 1. Crystallographic data for capsule structure

**Structure, CCDC no**	**capsule, 1469874**
Empirical formula	$C_{75}H_{63}N_3O_9S_3$
Mol. Weight / g mol^{-1}	1246.46
Temperature / *K*	100(2)
Wavelength / Å	0.6889
Crystal system	Tetragonal
Space group	$P4_22_12$
a / Å	29.528(2)
b / Å	29.528(2)
c / Å	42.498(3)
α / °	90
β / °	90
γ / °	90
Volume / Å^3	37055(6)
Z	16
Density / g cm^{-3}	0.894
Absorption Coeff. / mm^{-1}	0.123
Crystal size / mm^3	0.21 x 0.18 x 0.14
Θ range / °	1.325 to 18.247°
Index ranges	-32 ≤ h ≤ 32<br>-32 ≤ k ≤ 32<br>-47 ≤ l ≤ 45
Reflections collected	258765
Independent reflections	26609 [R(int) = 0.1756]
Completeness	99.8 % (to Θ = 22.502°)
Absorption correction	Semi-empirical from equivalents
Max. & min. transmission	0.7451 and 0.5094
Data / restraints / param.	26609 / 5526 / 2269
Goodness-of-fit on F^2	1.137
Final *R* indices [I > 2 s (I)]	R1 = 0.1025, wR2 = 0.2818
R indices (all data)	R1 = 0.1398, wR2 = 0.3127
Extinction coefficient	-
Larg. diff. peak/hole / eÅ^{-3}	0.510 and -0.388
Flack x (Parsons)	0.08(3)

Supplementary Table 2. Hydrogen bonds for capsule structure

D-H...A	d(D-H) [**Å**]	d(H...A) [**Å**]	d(D...A) [**Å**]	<(DHA) [**°**]
N8_1-H8_1...O11_1#3	0.88	2.05	2.888(12)	160
O12_1-H12_1...O38_1#3	0.84	1.83	2.669(11)	172
N38_1-H38_1...O11_2#1	0.88	2.15	2.998(8)	163
O42_1-H42_1...O68_2C#1	0.84	1.60	2.360(15)	149
O42_1-H42_1...O68_2D#1	0.84	2.04	2.874(17)	169
N68_1-H68_1...O41_2	0.88	2.13	3.007(12)	172
O72_1-H72_1...O8_2	0.84	1.75	2.572(10)	165
N8_2-H8_2...O11_2	0.88	2.40	2.708(10)	101
N8_2-H8_2...O41_1#1	0.88	2.29	3.161(8)	172
O12_2-H12_2...O68_1#1	0.84	1.75	2.566(10)	162
N38_2-H38_2...O71_1	0.88	2.14	3.006(11)	166
O42_2-H42_2...O8_1	0.84	1.83	2.656(11)	169
N68_2C-H68_2C...O71_2D#2	0.88	2.06	2.93(2)	170
O72_2C-H72_2C...O38_2#2	0.84	2.04	2.871(18)	169
N68_2D-H68_2D...O71_2C#2	0.88	2.13	2.98(2)	161
O72_2D-H168...O38_2#2	0.84	1.76	2.533(14)	153
C2_1-H2_1...N8_1	0.95	2.54	2.858(12)	100
C2_1-H2_1...O11_1#3	0.95	2.33	3.267(12)	170
C4_1-H4_1...O11_2#1	0.95	2.43	3.361(11)	167
C6_1-H6_1...O41_2	0.95	2.30	3.183(12)	153
C9_1-H9_1...O8_1	1.00	2.45	2.810(11)	101
C11_1-H11A_1...O11_1	0.99	2.50	3.351(15)	144
C25_1-H25_1...S12_1	0.95	2.62	3.071(9)	109
C39_1-H39_1...O38_1	1.00	2.30	2.745(11)	106
C41_1-H41A_1...O42_1	0.99	2.55	2.920(16)	102
C61_1-H61_1...S42_1	0.95	2.68	3.131(12)	110

C69_1-H45...O68_1	1.00	2.23	2.691(11)	106
C69_1-H69A_1...O68_1	1.00	2.30	2.691(11)	102
C75_1B-H75_1B...S72_1B	0.95	2.58	3.052(15)	111
C81_1B-H81_1B...S72_1B	0.95	2.72	3.069(16)	103
C87_1B-H87_1B...S42_2B	0.95	2.75	3.628(17)	155
C76_1-H76_1...O8_1	0.95	2.40	3.323(17)	163
C91_1-H91_1...S72_1B	0.95	1.82	2.539(16)	130
C91_1-H91_1...S72_1	0.95	2.72	3.15(2)	108
C2_2-H2_2...O71_1	0.95	2.29	3.189(12)	158
C4_2C-H4_2C...N68_2D	0.95	2.29	2.79(2)	112
C4_2C-H4_2C...O71_2D#2	0.95	2.07	2.97(2)	156
C6_2C-H6_2C...O68_2C	0.95	2.39	2.76(2)	103
C6_2C-H6_2C...O41_1#1	0.95	2.37	3.105(19)	134
C27_2-H27_2...S12_2	0.95	2.66	3.105(13)	110
C39_2-H39_2...O38_2	1.00	2.41	2.770(12)	100
C49_2A-H49_2A...S42_2A	0.95	2.69	3.087(16)	106
C49_2A-H49_2A...S42_2B	0.95	2.47	3.029(17)	117
C47_2B-H47_2B...O72_2C#2	0.95	2.25	2.80(2)	116
C55_2B-H55_2B...S42_2B	0.95	2.65	2.982(17)	101
C57_2B-H57_2B...S42_2B	0.95	2.61	3.063(18)	110
C69_2C-H69_2C...O68_2C	1.00	2.33	2.746(18)	104
C81_2C-H81_2C...S72_2C	0.95	2.61	3.075(18)	111
C4_2D-H4_2D...N68_2D	0.95	2.42	2.79(2)	102
C4_2D-H4_2D...O71_2C#2	0.95	2.47	3.42(2)	176
C4_2D-H4_2D...O71_2D#2	0.95	1.88	2.78(2)	156
C6_2D-H6_2D...O68_2C	0.95	2.28	2.78(2)	112
C6_2D-H6_2D...O41_1#1	0.95	2.36	3.149(19)	141
C69_2D-H69_2D...S72_2C	1.00	2.69	3.215(15)	113

C69_2D-H69_2D…O68_2D	1.00	2.31	2.74(2)	104
C75_2D-H75_2D…S72_2D	0.95	2.64	3.00(2)	103
C87_2D-H87_2D…S72_2D	0.95	2.75	3.112(17)	104
C2_2-H2_2…O71_1	0.95	2.29	3.189(12)	158
C4_2C-H4_2C…N68_2D	0.95	2.29	2.79(2)	112
C4_2C-H4_2C…O71_2D#2	0.95	2.07	2.97(2)	156
C6_2C-H6_2C…O68_2C	0.95	2.39	2.76(2)	103
C6_2C-H6_2C…O41_1#1	0.95	2.37	3.105(19)	134
C27_2-H27_2…S12_2	0.95	2.66	3.105(13)	110
C39_2-H39_2…O38_2	1.00	2.41	2.770(12)	100
C49_2A-H49_2A…S42_2A	0.95	2.69	3.087(16)	106
C49_2A-H49_2A…S42_2B	0.95	2.47	3.029(17)	117
C47_2B-H47_2B…O72_2C#2	0.95	2.25	2.80(2)	116
C55_2B-H55_2B…S42_2B	0.95	2.65	2.982(17)	101

Symmetry transformations used to generate equivalent atoms:

#1 = 1-y,1-x,1-z , #2 = 2-x,-y,z , #3 = 1+y,-1+x,1-z

Atom label details:

_1 = Residue 1

_2 = Residue 2

_1A = Residue 1, Part 1

_1B = Residue 1, Part 2

_2A = Residue 2, Part 1

_2B = Residue 2, Part 2

_2C = Residue 2, Part 3

_2D = Residue 2, Part 4

Supplementary methods

General

Chemicals and solvents were purchased from commercial suppliers and used as received. NMR spectra were recorded on Bruker Fourier 300 MHz (5mm Fourier 1H/13C probe), Bruker Avance III HD 400 MHz (5 mm BBFO probe), Bruker 500 MHz Avance III HD (5 mm TXI probe), Bruker Avance III HD 600 MHz (5 mm BBFO probe) or Bruker Avance III HD 700 MHz (5 mm BBI probe) spectrometers. Spectra were referenced on the solvent residual peaks. NMR solvents were purchased from Euriso-Top or Deutero GmbH and used as received. UV-Vis absorption spectra were recorded on Thermo Fisher Scientific Evolution 201 spectrophotometer in Hellma quartz cuvette at path length d=2 mm. Baseline was recorded on the free solvent. Circular Dichroism (CD) spectra were recorded on Jasco J-810 spectropolarimeter in Hellma quartz cuvette at path length d=1 mm. Baseline was recorded on the free solvent. Spectra were smoothed using Savitzky-Golay filter. MALDI MS TOF/TOF spectra were recorded on Bruker ultrafleXtreme™ mass spectrometer using DCTB matrix (Sigma-Aldrich cat. 87884).

Synthesis

Synthesis of benzene-1,3,5-tricarboxylic acid NHS ester: To a solution of benzene-1,3,5-tricarboxylic acid (0.78 g, 3.70 mmol) in anhydrous DMF (20 mL), *N*-hydroxysuccinimide (2.55 g, 22.2 mmol) and 1-ethyl-3-(3-dimethylaminopropyl)-carbodiimide hydrochloride (4.26 g, 22.2 mmol) were added. The mixture was stirred at room temperature for 24 h under a nitrogen atmosphere. After solvent removal, the residue was redissolved in acetone (25 mL) and poured into 1M HCl (200 mL). The white precipitate was filtered off, washed with H2O (50 mL) and hot isopropanol (50 mL) and dried under high vacuum (70 % yield). ^{1}H-NMR (500 MHz, DMSO-d$_6$) δ(ppm): 8.93 (s, 1H), 2.92 (s, 4H); ^{13}C NMR (125.75 MHz, DMSO-d$_6$) δ(ppm): 170.33, 160.12, 136.72, 127.77, 25.99.

*Synthesis of L-**1**:* To a solution of A (0.50 g, 0.99 mmol) in anhydrous DMF (35 mL), S-Trityl-L-cysteine (2.17 g, 5.98 mmol) and triethylamine (0.60 g, 5.98 mmol) were added. The mixture was stirred at room temperature for 24 h under a nitrogen atmosphere. After solvent removal, the residue was redissolved in acetone (15 mL) and poured into 1M HCl (150 mL). The light yellow precipitate was filtered off, washed with H_2O (50 mL) and dried under high vacuum (78 % yield). ^{1}H NMR (300 MHz, DMSO-*d$_6$*) δ 12.82 (bs, 3H), 9.15 (d, J = 8.0 Hz, 3H), 8.50 (s, 3H), 7.35 – 7.29 (m, 36H), 7.28 – 7.20 (m, 9H), 4.36 (td, J = 9.0, 4.7 Hz, 3H), 2.86 – 2.75 (m, 3H), 2.56 (dd, J = 12.7, 4.7 Hz, 3H). ^{13}C NMR (75 MHz, DMSO-*d$_6$*) δ 171.54, 165.38, 144.26, 134.32, 129.32, 129.12, 128.11, 126.84, 66.39, 52.34, 32.69. MALDI MS: Calc. $[M-H]^-$= 1244.365 m/z. Found: $[M-H]^-$ 1244.963, $[2M-2H+K]^-$ 2529.990 m/z.

*Synthesis of D-**1**:* D-**1** was synthesised according to L-1 procedure using S-Trityl-D-cysteine. (74% Yield). ^{1}H NMR (300 MHz, DMSO-*d$_6$*) δ 12.80 (bs, 3H), 9.13 (d, J = 8.0 Hz, 3H), 8.52 (s, 3H), 7.35 – 7.28 (m, 36H), 7.28 – 7.20 (m, 9H), 4.35 (td, J = 9.0, 4.7 Hz, 3H), 2.86 – 2.75 (m, 3H), 2.54 (dd, J = 12.7, 4.7 Hz, 3H). ^{13}C NMR (75 MHz, DMSO-*d$_6$*) δ 171.52, 165.37, 144.24, 134.30, 129.31, 129.11, 128.09, 126.82, 66.37, 52.34, 32.67. MALDI MS: Calc. $[M-H]^-$= 1244.365 m/z. Found: $[M-H]^-$ 1244.963, $[2M-H]^-$ 2491.147 m/z.

*Synthesis of L-**2**:* L-**2** was synthesised according to L-1 procedure using S-Trityl-L-cysteine *tert*-butyl ester. (81% Yield) ^{1}H NMR (300 MHz, DMSO-d_6) δ 9.15 (d, *J* = 7.8 Hz, 3H), 8.49 (s, 3H), 7.36 – 7.29 (m, 36H), 7.25 (m, 9H), 4.23 (m, 3H), 2.81 (t, *J* = 11.2 Hz, 3H), 2.44 (dd, *J* = 12.8, 4.7 Hz, 3H), 1.30 (s, 27H). ^{13}C NMR (75 MHz, DMSO-d_6) δ 169.13, 165.53, 144.19, 134.32, 129.35, 129.11, 128.06, 126.80, 81.05, 66.58, 53.29, 32.68, 27.51. MALDI MS: Calc. $[M+K]^+$= 1452.523 m/z. Found: $[M+K]^+$= 1452.960 m/z.

Single-Crystal X-ray diffraction

Crystallographic data are summarized in Supplementary Tables 1 and 2. Crystals of L-**1** needed to be picked quickly and flash cooled in the nitrogen cold gas stream immediately after preparation. Due to their limited scattering power, home sources failed to provide sufficient resolution to solve the structure. Data were collected at Beamline I19 of Diamond Light Source[2] employing silicon double crystal monochromated synchrotron radiation (0.6889 Å) with ω scans at 100(2) K. Frames were converted to Bruker format (.sfrm) and data integration were undertaken with SAINT[3]. Multi-scan empirical absorption correction was applied to the data using SADABS[3]. Although using a highly brilliant 3rd generation synchrotron source the scattering power was not sufficient to fulfill small molecule resolution criteria (0.84 Å) for data quality in terms of resolution requirements and internal R value (Rint). When using all reflections up to 0.84 Å, space group determination and structure solution attempts were unsuccessful in our hands. For reducing the amount of noise in the dataset a resolution cut off at 1.1 Å was performed to achieve a mean I/sig(I) > 3 in the highest resolution shell. This allowed for a successful space group determination using XPREP[3] and to subsequently solve the structure in the macromolecular direct methods program SHELXDD[4] and refined with SHELXL[5] using full-matrix least-squares routines on F^2 and ShelXle[6] as a GUI (Graphical User Interface) .

To facilitate structure refinement and molecular model building, a series of macromolecular refinement techniques has been carefully adapted and employed. These methods already proved successful in previous cases of huge and complicated supramolecular structures with high solvent content.[7, 8, 9] L1 ligands were grouped into residues to enable addressing all the atoms of repeating structural fragments with a single command. Stereochemical restraint dictionaries were generated using GRADE. GRADE is part of BUSTER[10] and was accessed via the GRADE Web Server.[11] Its dictionaries for SHELXL contain target values and standard deviations for 1,2-distances (DFIX) and 1,3-distances (DANG), as well as restraints for planar groups (FLAT). GRADE restraint dictionaries of disordered moieties were loaded into the database of DSR[12]. DSR was subsequently employed to place and refine those disordered moieties in a semi-automatic fashion by starting with a rigid body refinement followed by a restrained refinement using restraints from GRADE dictionaries. The refinement of ADPs for all non-hydrogen atoms was enabled by employing similarity (SIMU), and enhanced rigid bond restraints (RIGU)[13] in the SHELXL program.[5] Carbon-bound hydrogen atoms were included in idealized positions and refined using a riding model. The contribution of the electron density associated with disordered counterions and solvent molecules, which could not be modelled with discrete atomic positions were handled using the SQUEEZE[14] routine in PLATON[15]. Solvent masks (.fab files) generated by PLATON were included in the SHELXL refinement via the ABIN instruction in order to leave the original structure factors untouched.

Crystallographic data including structure factors have been deposited with CCDC no. 1469874. Copies of the data can be obtained free of charge at the CCDC website http://www.ccdc.cam.ac.uk/structures.

Cavity Volume Calculation

The capsule structure was symmetry expanded to P1 and half of the disordered trityl substituents were removed. The resulting inner cavity volume was calculated with VOIDOO [16] using a primary grid and plot grid spacing of 0.2 Å and ten cycles of volume refinement. To prevent the probe from "escaping" the inner sphere through the large pores, the default water size probe radius of 1.4 Å was increased to 3.0 Å. This results in smaller calculated volumes compared to using the default probe size, but provides the additional information that all molecules with diameter of 2.9 Å or smaller can penetrate the capsule. Molecular visualizations were done using PyMol[17].

Binding constant determination

The equilibrium of host-guest complexation can be expressed as follows:

$$[H]+[G]\rightleftarrows[HG]$$

The binding constant K is expressed as:

$$K=([HG])/([H]\times[G])$$

Host-guest binding constants were obtained from UV-Vis titration of free fullerenes (guest) with L-1 (host) from 0 to 14 equiv., using Benesi-Hildebrand method (Supplementary Figure 28), and cross checked with binding constans obtained from ^{13}C NMR spectra (Supplementary Figures 13, 15) where [G], and [HG] signals were obtained separatelly allowing determination of the approx. K value directly from the equation above.

UV-Vis titration details

Stock solutions of L-capsule, C60, and C70 were prepared in TCE at C = 1x10^{-3} M. Fullerenes (guests) working solutions were obtained by dilution of stock solutions to C = 5x10-5 M with TCE. In order to keep the guest concentration constant during titration, the fullerenes working solutions were titrated with solutions containing: capsule at C = 5x10^{-4} M and corresponding fullerene at C = 5x10$^{-5\ M}$, which one was also obtained by dilution of stock solutions with TCE.

Supplementary References

1. Stefankiewicz A. R., Sambrook M. R., Sanders J. K. M. Template-directed synthesis of multi-component organic cages in water. Chem. Sci. **3**, 2326-2329 (2012).

2. Nowell H., Barnett S. A., Christensen K. E., Teat S. J., D. R. Allan. J. Synchrotron Rad. **19**, 435-441. (2012).

3 Bruker-Nonius, APEX, SAINT, SADABS and XPREP, Bruker AXS Inc., Madison, Wisconsin, USA (2013).

4. Sheldrick G. M. A short history of SHELX. Acta Cryst. A **64**, 112-122 (2008).

5. Sheldrick G. M. Crystal structure refinement with SHELXL. Acta Cryst. C **71**, 3–8 (2015).

6. Hübschle C. B., Sheldrick G. M., Dittrich B. ShelXle: a Qt graphical user interface for SHELXL. J. Appl. Cryst. **44**, 1281–1284 (2011).

7. Pascu M., Marmier M., Schouwey C., Scopelliti R., Holstein J. J., Bricogne G., Severin K., Anionic Bipyridyl Ligands for Applications in Metallasupramolecular Chemistry. Chem. Eur. J. **20**, 5592–5600 (2014).

8. Schouwey C., Holstein J. J., Scopelliti R., Zhurov K. O., Nagornov K. O., Tsybin Y. O., Smart O. S., Bricogne G., Severin K. Self-assembly of a giant molecular Solomon link from 30 subcomponents. Angew. Chem. Int. Ed. **53**, 11261 –11265 (2014).

9. Ronson T. K., Giri C., Beyeh N. K., Minkkinen A., Topić F., Holstein J. J., Rissanen K., Nitschke J. R. Size-Selective Encapsulation of Hydrophobic Guests by Self-Assembled M4L6 Cobalt and Nickel Cages. Chem. Eur. J. **19**, 3374–3382 (2013).

10 Bricogne G., Blanc E., Brandl M., Flensburg C., Keller P., Paciorek P., Roversi P., Sharff A., Smart O., Vonrhein C., Womack T. BUSTER version 2.13.0, 2011, Global Phasing Ltd., Cambridge, United Kingdom.

11 http://grade.globalphasing.org.

12. A. Thorn, B. Dittrich and G. M. Sheldrick. Enhanced rigid-bond restraints. Acta Cryst. A **68**, 448–451 (2012).

13. Kratzert, D., Holstein, J.J. & Krossing, I. DSR: enhanced modelling and refinement of disordered structures with SHELXL. J. Appl. Cryst. **48**, 933-938 (2015).

14. van der Sluis P., Spek A. L. BYPASS: an effective method for the refinement of crystal structures containing disordered solvent regions Acta Cryst. A **46**, 194–201 (1990).

15. Spek A. L. Structure validation in chemical crystallography. Acta Cryst. D **65**, 148-155 (2009).

16. Kleywegt G. J., Jones T. A. Detection, delineation, measurement and display of cavities in macromolecular structures. Acta Cryst. D **50**, 178–185, (1994).

17. DeLano W. L. The PyMol Molecular Graphics System. DeLano Scientific LLC, San Carlos, CA (USA).
